# The genomic landscape of the verrucomicrobial methanotroph *Methylacidiphilum fumariolicum* SolV

**DOI:** 10.1186/1471-2164-15-914

**Published:** 2014-10-20

**Authors:** Seyed Yahya Anvar, Jeroen Frank, Arjan Pol, Arnoud Schmitz, Ken Kraaijeveld, Johan T den Dunnen, Huub JM Op den Camp

**Affiliations:** Department of Human Genetics, Leiden University Medical Center, Leiden, The Netherlands; Leiden Genome Technology Center, Leiden University Medical Center, Leiden, The Netherlands; Department of Microbiology, Radboud University, Nijmegen, The Netherlands; Department of Ecological Science, VU University Amsterdam, Amsterdam, The Netherlands; Department of Clinical Genetics, Leiden University Medical Center, Leiden, The Netherlands

**Keywords:** *Methylacidiphilum fumariolicum* SolV, Genome assembly, Single molecule sequencing, Pacific biosciences, Methylation, Gene expression, Verrucomicrobial methanotrophs

## Abstract

**Background:**

Aerobic methanotrophs can grow in hostile volcanic environments and use methane as their sole source of energy. The discovery of three verrucomicrobial *Methylacidiphilum* strains has revealed diverse metabolic pathways used by these methanotrophs, including mechanisms through which methane is oxidized. The basis of a complete understanding of these processes and of how these bacteria evolved and are able to thrive in such extreme environments partially resides in the complete characterization of their genome and its architecture.

**Results:**

In this study, we present the complete genome sequence of *Methylacidiphilum fumariolicum* SolV, obtained using Pacific Biosciences single-molecule real-time (SMRT) sequencing technology. The genome assembles to a single 2.5 Mbp chromosome with an average GC content of 41.5%. The genome contains 2,741 annotated genes and 314 functional subsystems including all key metabolic pathways that are associated with *Methylacidiphilum* strains, including the CBB pathway for CO_2_ fixation. However, it does not encode the serine cycle and ribulose monophosphate pathways for carbon fixation. Phylogenetic analysis of the particulate methane mono-oxygenase operon separates the *Methylacidiphilum* strains from other verrucomicrobial methanotrophs. RNA-Seq analysis of cell cultures growing in three different conditions revealed the deregulation of two out of three *pmoCAB* operons. In addition, genes involved in nitrogen fixation were upregulated in cell cultures growing in nitrogen fixing conditions, indicating the presence of active nitrogenase. Characterization of the global methylation state of *M. fumariolicum* SolV revealed methylation of adenines and cytosines mainly in the coding regions of the genome. Methylation of adenines was predominantly associated with 5′-^**m6**^**A**CN_4_GT-3′ and 5′-CC^**m6**^**A**N_5_CTC-3′ methyltransferase recognition motifs whereas methylated cytosines were not associated with any specific motif.

**Conclusions:**

Our findings provide novel insights into the global methylation state of verrucomicrobial methanotroph *M. fumariolicum* SolV. However, partial conservation of methyltransferases between *M. fumariolicum* SolV and *M. infernorum* V4 indicates potential differences in the global methylation state of *Methylacidiphilum* strains. Unravelling the *M. fumariolicum* SolV genome and its epigenetic regulation allow for robust characterization of biological processes that are involved in oxidizing methane. In turn, they offer a better understanding of the evolution, the underlying physiological and ecological properties of SolV and other *Methylacidiphilum* strains.

**Electronic supplementary material:**

The online version of this article (doi:10.1186/1471-2164-15-914) contains supplementary material, which is available to authorized users.

## Background

The discovery of three verrucomicrobial methanotrophs that constitute the *Methylacidiphilum* genus [1–4] and characterization of their ecological, physiological, and phylogenetic properties have shed light on the diversity of processes through which aerobic methanotrophs use methane as their sole source of carbon and energy [5]. A remarkable characteristic of these bacteria is their ability to oxidize methane in extreme and hostile conditions of volcanic and geothermal areas. Three *Methylacidiphilum* strains (*M. fumariolicum* SolV, *M. kamchatkense* Kam1, *M. infernorum* V4) were isolated from acidic volcanic areas in Italy, Russia, and New Zealand, respectively [1–3]. The draft genome assembly of *M. fumariolicum* SolV and the complete genome sequence of *M. infernorum* V4 have previously been published [6, 7], showing over 98% sequence identity for their 16S rRNA genes [4]. Likewise, phylogenetic analysis of the *pmoA* genes, encoding the 24 kDa β-subunit of particulate methane mono-oxygenase (pMMO), revealed a strong similarity of these strains and their separation from other methanotrophs [3]. In addition, major differences in C1 utilization pathways were found between these strains and other proteobacterial and NC10 methanotrophs [8]. A comprehensive understanding of how these bacteria have evolved and thrive in such hostile environmental conditions partially relies on deciphering their genetic diversity and architecture.

The draft genome of *M. fumariolicum* SolV was previously constructed using Illumina GAII and Roche 454 reads [6]. Despite the high coverage of Illumina GAII and Roche 454 sequencing reads as well as improvement of the assembly by manual curation of the assembly graph, the genome of *M. fumariolicum* SolV remained fragmented (109 contigs and a N50 value of 50,138 bp). The short lengths of Illumina GAII and Roche 454 sequencing reads can prevent the assembler from resolving repeats, which leaves the assembly incomplete. Furthermore, regions with high or low GC content are difficult to PCR and thus to sequence using second-generation sequencing platforms. Here, we report the complete genome sequence of the *M. fumariolicum* SolV that was determined by Pacific Biosciences single-molecule real-time (SMRT) sequencing technology. Using SMRT sequencing, long and highly accurate single-molecule sequencing reads were generated to resolve long repeats that remained in the unfinished and fragmented draft genome. These reads can resolve regions with extreme GC content, palindromic sequences, and other sequence contexts that challenge other sequencing platforms. Following the completion of the genome and the annotation of genes and functional subsystems, we characterize the phylogenetic relationship between the genome of *M. fumariolicum* SolV and that of other methanotrophs, particularly the *M. infernorum* V4. In order to assess the accuracy of the single chromosome assembly, two independent SMRT sequencing runs were generated and aligned to determine the consensus accuracy. Next, we performed genome-wide expression analysis to understand how major pathways are regulated in *M. fumariolicum* SolV cell cultures grown under different conditions. Bacterial DNA methylation is believed to play a role in maintaining genome integrity, gene regulation, and as a defence mechanism to identify and destroy foreign DNA that is differentially methylated [9–12]. So far, the global methylation state of bacterial kingdom is poorly understood [13], owing to the complexity and laborious nature of experiments that are needed for such studies. In the advent of SMRT sequencing technology, polymerase kinetics during sequencing can be used to identify N6-methladenine (6mA), 4-methylcytosine (4mC), and 5-methylcytosine (5mC) at a base-pair resolution [14]. Since *M. fumariolicum* SolV genome contains copies of methyltransferases, we extend the analysis to epigenetic characterization of *M. fumariolicum* SolV by providing a global methylation state of the genome and the associated motifs. Finally, we explore the occurrence of methylation within the coding sequences that can affect the regulation of genes.

## Results

### Genome assembly and annotation

To obtain the complete genome sequence of *M. fumariolicum* SolV, eight SMRT sequencing runs were performed that yielded 234,459 long (300 bp to 23,000 bp) and high-quality single-molecule sequencing reads (Additional file 1: Figure S1). Despite the high coverage of the single-molecule sequencing reads, the presence of randomly distributed sequencing errors prevents *de novo* assembly on filtered subreads. A number of strategies have been proposed to correct single-molecule sequencing reads [15–17]. In order to avoid introducing inherent biases of second-generation sequencing technologies and to take full advantage of single-molecule sequencing reads, we have used hierarchical genome-assembly process (HGAP) to correct sequencing errors in filtered subreads. HGAP [16] relies on shorter single-molecule sequencing reads to construct highly accurate preassemblies on single-molecule sequencing reads that are longer than the seed length. The HGAP pipeline, using a seed length of 1,500 bp, resulted in 48,452 corrected reads that ranged between 501 bp and 15,852 bp in length and had an average GC content of 41.7% (Additional file 1: Figure S2). Despite the significant loss of sequencing depth during the correction procedure (Table 1), sufficient coverage depth (36×) remained to perform a *de novo* assembly using the overlap-layout-consensus (OLC) strategy.

**Table 1:** **Read statistics of 8 SMRT sequencing runs pre and post correction**

	PacBio RS (raw)	PacBio RS (corrected)*
Number of reads	234,459	48,452
Total nucleotides	352,940,647	90,484,833
Median read length	1,263 bp	1,742 bp
5^th^ percentile	396 bp	699 bp
95^th^ percentile	3,374 bp	3,311 bp
Maximum length	22,910 bp	15,852 bp
GC content	43.54%	41.70%
Coverage depth	141.74×	36.34×

*Error-corrected PacBio reads generated by HGAP with seed length of 1,500 bp.

**Figure 1:**
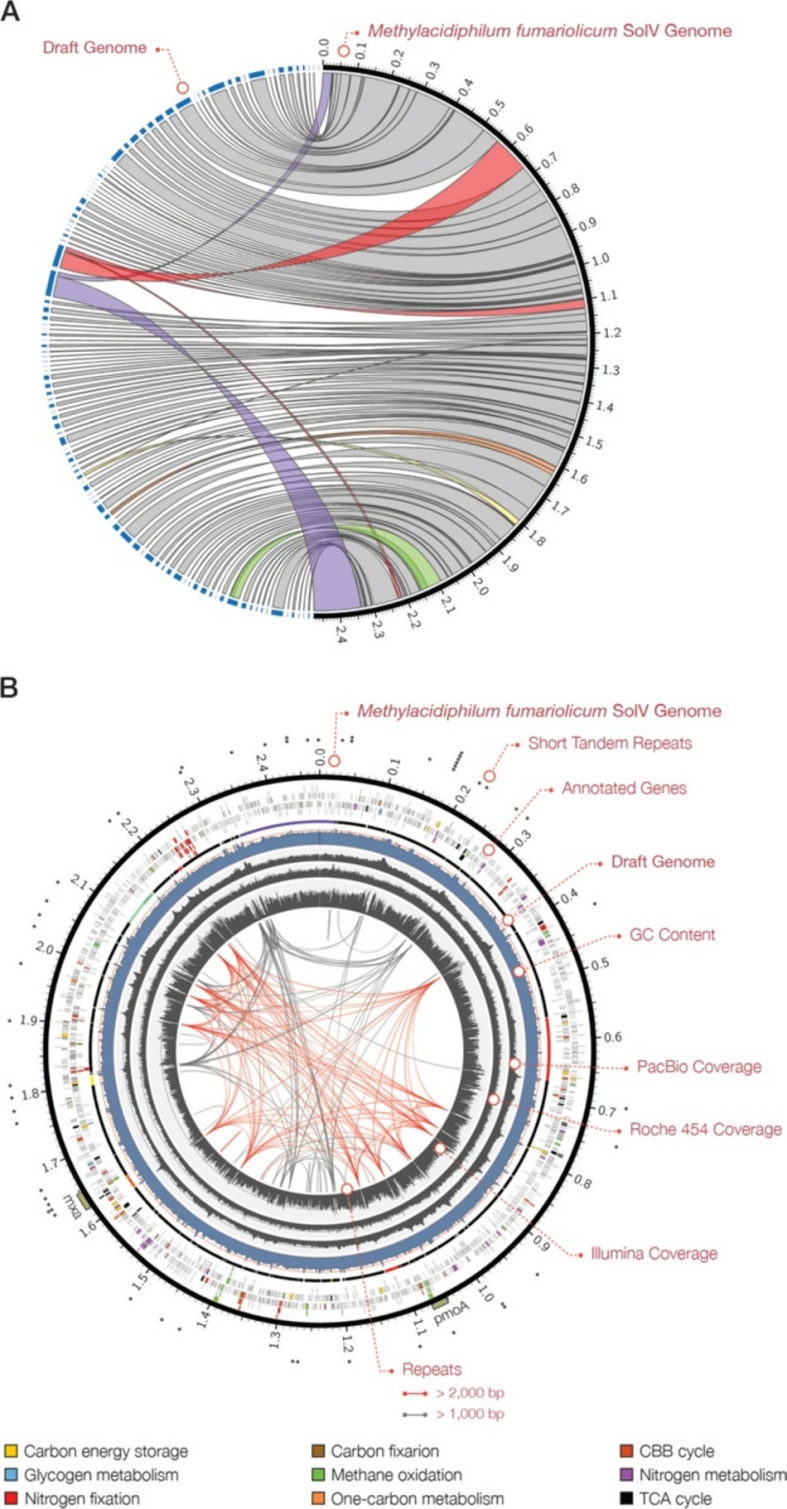
**The** ***Methylacidiphilum fumariolicum*** **SolV genome sequence. A)** Circos plot depicts the level of concordance between draft genome and the final assembly. Coloured links highlight misassemblies in the draft genome. **B)** Circos plot illustrates the overall genetic makeup of the *Methylacidiphilum fumariolicum* SolV. The outer ring marks the positions of tandem repeats across the genome. The next rings (outside to inside) show: gene annotation, highlighting key biological pathways in colours; placement of the draft genome in respect to the final assembly; and the overall GC content and the coverage profile of SMRT, Roche 454, and Illumina GAII sequencing reads. Repetitive sequences and structural variations are linked across the genome. Repeats that are longer than 2 Kb are shown in red whilst shorter repeats are linked in grey.

Celera Assembler 7.0 was used to assemble the corrected single-molecule sequencing reads into a single contig. The complete genome sequence of *M. fumariolicum* SolV is 2,476,673 base pairs in length with an average GC content of 41.5% (Table 2). Compared to the draft genome sequence [6], the final assembly contains 114,257 more bases and has a 0.57% higher GC content. We identified four misassembled contigs in the draft genome that could be split and mapped to different genomic locations (Figure 1A). Misassemblies were the result of the presence of repeats, small close-range duplications, and lower coverage. In addition, we identified CAHT01000038.1 as the only contig that could not be mapped (partially or fully) to the final assembly. The presence of this sequence in the genome of *M. fumariolicum* SolV could not be supported by the alignment of long reads to the draft genome. The only strong BLAST hit for the CAHT01000038.1 sequence (1,086 bp) was to the mitochondrial genome of *Cygnus columbianus (bewickii)*. The total of 93 gaps in the draft genome summed to a total of 110,521 bp. Although, on average, these sequence gaps were rather short (median =1,157 bp), they had a very high GC content of 53.46% compared to that of the entire genome (Additional file 1: Figure S3). The 11.98% increase in GC content is in concordance with known limitation of second generation sequencing technologies in sequencing genomic regions with extreme GC contents.

**Table 2:** **SMRT** ***de novo*** **genome assembly statistics**

	Draft genome ^1^	SMRT de novo ^2^
Number of reads	16,099,262	48,452
Sequencing depth	401.23×	36.34×
Number of contigs	109	1
Bases in scaffolds	2,362,416 bp	2,476,673 bp*
N50	50,138 bp	2,476,673 bp
Maximum length	166,468 bp	2,476,673 bp
GC content	40.91%	41.48%
Genome coverage	95.54%**	100%***
Accuracy	99.9958%	99.9998%

^1^Draft genome assembled using Illumina GAII and Roche 454 reads using CLCBio (CLCBio, Aarhus, Denmark) and curated manually [6].

^2^SMRT *de novo* assembly was carried out on corrected PacBio reads using Celera Assembler 7.0.

*The total bases in the scaffolds were determined after circularization of the final assembly.

**The overall genome coverage is determined by calculating the total number of gaps in the draft genome as compared to the final assembly.

***The genome coverage of SMRT *de novo* is determined by aligning PacBio reads, generated by two independent SMRT sequencing runs, to the final assembly.

The accuracy of the final assembly was assessed after aligning single-molecule sequencing reads that were generated from two independent SMRT sequencing runs. We observed a consensus accuracy of 99.9998% between reads and the reference sequence, with no significant coverage fluctuation over the entire genome. Moreover, 99.9% of Illumina GAII and Roche 454 reads (Additional file 1: Table S1, Figures S4 and S5) that were used to assemble the draft genome [6] mapped to the complete genome sequence (Figure 1B). However, we observed significant fluctuations in Illumina GAII coverage that often coincides with gaps in the draft genome and the presence of repeats. We found 409 repeats across the genome, including 54 short tandem repeats (STRs). We observed a high GC content (55.5%) across larger repeats (median length of 1,826 bp), whereas tandem repeats had a low GC content of 27.9% (Additional file 1: Figure S6). This is not the case for the genome of *M. infernorum* V4 as only a few short tandem and larger repeats were found (Additional file 1: Figures S7 and S8). The co-occurrence of repeats and low depth of coverage can significantly hamper the assembly and may explain the fragmentation of the draft genome.

After annotating the complete genome sequence using RAST [18], we identified 2,741 protein-encoding genes and 49 RNAs, from which 932 (33.4%) were allocated to 314 annotated subsystems, biological processes or structural complexes that are realised by a set of functional roles [19]. The origin of replication was identified by GC Skew analysis [20] and mapped to approximately 6,597 nucleotides upstream of the *dnaA* gene that is located at 326002–327357 genomic coordinates. The terminus of DNA replication is approximately located at 1,450,937 genomic position. The complete genome annotation contains 458 newly discovered genes, from which 178 were found in gaps in the draft genome. Only 11.2% of genes that are fully or partially located in gaps had known function whereas the remaining genes were annotated as hypothetical proteins (Additional file 1: Table S2). We also identified two newly annotated genes (PEG.1144 and PEG.1150) that belong to the C-subunit of pMMO that were absent in the draft assembly. Furthermore, the annotations of genes that belong to key metabolic pathways were manually curated based on comparison to public databases (Figure 1B).

### Phylogenetic and comparative genome analysis

Except for the facultative methanotroph *Methylocella tundrae* [21] and the obligate methanotroph *Methyloferula stellata* [22], all aerobic methanotrophs known so far contain a membrane-bound particulate methane mono-oxygenase (pMMO). Therefore, the *pmoA* gene (encoding the ~24 kDa β-subunit of pMMO) has been widely used as a marker to determine the phylogeny of methanotrophic bacteria, which is largely comparable to that of the 16S rRNA-based phylogeny [23–26]. The phylogenetic relationship between annotated PmoA proteins indicates a strong separation of the genus *Methylacidiphilum* (consisting of *M. fumariolicum* SolV, *M. infernorum* V4 [7], and *M. kamchatkense* Kam1 [2]) from other methanotrophs (Additional file 1: Figure S9). The *pmoA3* gene shows a very distinctive branch in the phylogenetic tree whereas *pmoA1* and *pmoA2* genes are clustered together as a separate *Methylacidiphilum* branch deep in the main cluster. These observations are in concordance with phylogenetic relationships that were previously reported for PmoA proteins [3, 4, 6].

Since *M. infernorum* V4 is the closest relative for which the complete genome sequence is known [7], we compared the genome of *M. fumariolicum* SolV to this strain. The analysis highlights a number of inversions and transpositions between two genomes (Additional file 1: Figure S10). Amino acid comparison of protein-encoding genes (PEGs) revealed that 24.4% of PEGs are present in the genome of *M. infernorum* V4 with more than 80% identity, whereas 32.6% were exclusive to *M. fumariolicum* SolV (Additional file 1: Figure S11). Notably, 64.3% of PEGs have at least 50% identity to the genome of *M. infernorum* V4 (Additional file 1: Table S3). Shared PEGs are distributed across the entire genome. In addition, we did not find a significant enrichment of PEGs that are exclusive to either genome in a specific metabolic pathway (Additional file 1: Figure S12).

### Transcriptome analysis

**Figure 2:**
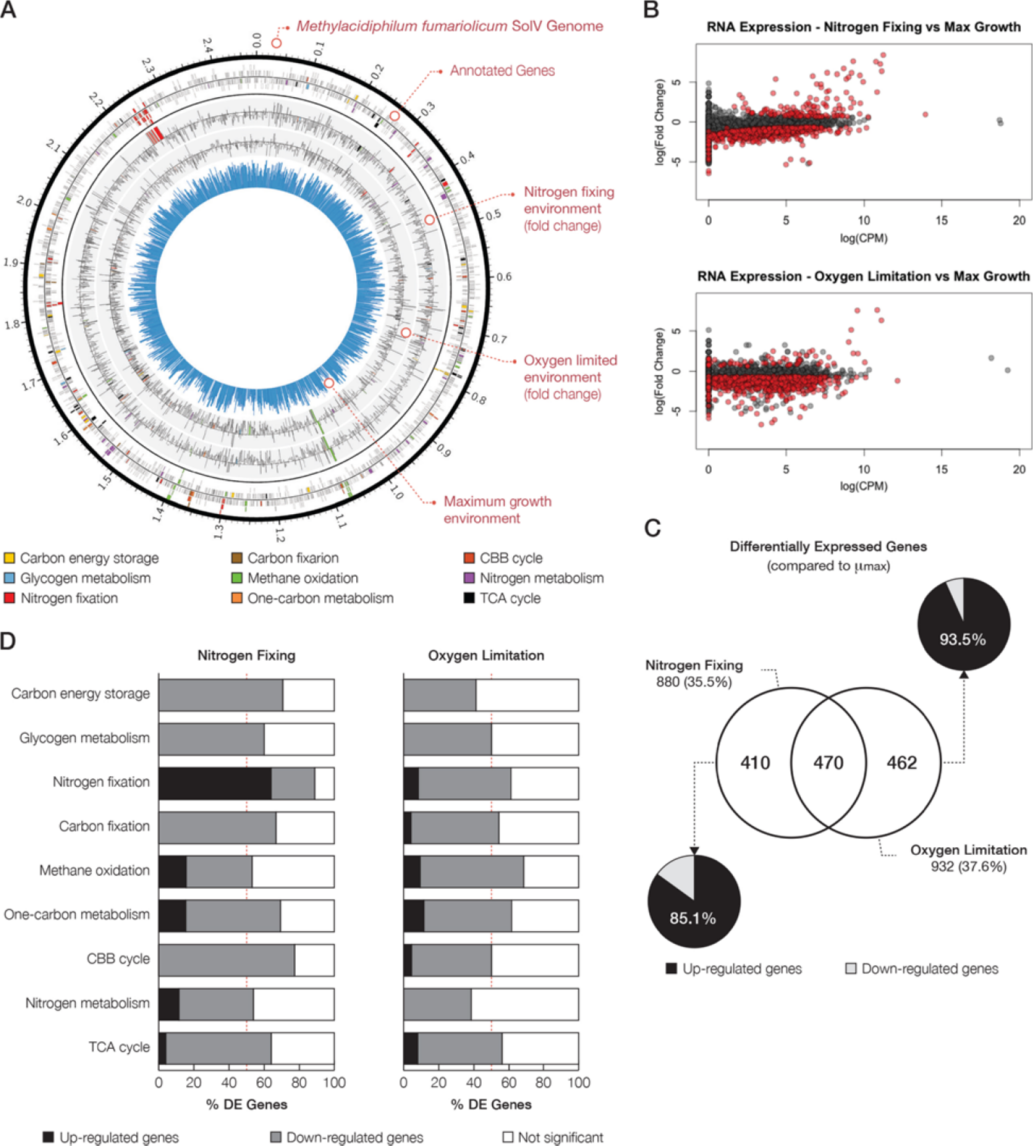
**Metabolic regulation of** ***Methylacidiphilum fumariolicum*** **SolV cell cultures grown under different conditions. A)** Circos plot depicts the genome-wide expression profile for cell cultures under maximum growth conditions (blue) and the relative gene expressions (fold change) of cell cultures grown under nitrogen fixation or oxygen limitation conditions. Count-per-million (CPM) was used to determine the level of gene expression. Key biological pathways are highlighted in different colours. **B)** MA plot for cell cultures under nitrogen fixation condition as compared to cell cultures in maximum growth environment. Deregulated genes are depicted in red. MA plot for cell cultures under oxygen limitation condition as compared to cell cultures in maximum growth environment. Deregulated genes are depicted in red. **C)** Venn diagram shows the number of genes that are differentially expression in both N_2_fix and O_2_lim conditions compared to μmax. Pie charts illustrate the fraction of genes that have a higher (black) or lower (light grey) expression in N_2_fix and O_2_lim cell cultures relative to μmax. **D)** Bar charts present the fraction of up- or down-regulated genes (black and light grey, respectively) in each of the nine key pathways. Red line depicts the 50% mark. The proportion of non-significant genes is depicted in white.

Cells cultured under three different conditions (μ_max_, N_2_ fixing, and O_2_ limited) were previously used to sequence mRNAs in three independent RNA sequencing (RNA-Seq) experiments [8]. The gene expression analysis was previously performed on the draft genome of *M. fumariolicum* SolV [8]. Here, we extend this analysis using the complete sequence and annotation of this genome. 19.1 × 10^6^, 18.9 × 10^6^, and 17.6 × 10^6^ single-end sequencing reads were generated for these cell cultures, respectively. Subsequently, reads were mapped to the complete genome of *M. fumariolicum* SolV and filtered for those that mapped to the ribosomal RNA operon. Over 99.8% of sequencing reads mapped to the reference sequence with concordance to the genome annotation. Next, RNA-Seq data from cell cultures under nitrogen fixing (N_2_fix) and oxygen limited (O_2_lim) conditions were compared to RNA-Seq data from cell cultures growing at μ_max_ (Figure 2A). In N_2_fix and O_2_lim cultures, 35.5% and 37.6% of genes were differentially expressed with 470 genes present in both conditions (Figure 2B,C). From 458 newly annotated genes, 108 and 167 genes were identified as differentially expressed in N_2_fix and O_2_lim cell cultures, respectively. Since the majority of these genes are not attributed to specific subsystems, we could not assess the enrichment of key pathways in this set.

The majority of differentially expressed genes showed a relatively lower level of expression in N_2_fix and O_2_lim cell cultures compared to μ_max_ (85.1% and 93.5%, respectively). The expression levels (count-per-million; CPM) and associated statistics are provided for the curated list of genes that are present in nine key pathways (Additional file 1: Tables S4–S12). Despite a substantial down-regulation of genes in N_2_fix cell cultures, 71.9% of genes that are involved in nitrogen fixation were significantly upregulated (Figure 2D and Additional file 1: Figure S13). This observation is in agreement with our previous physiological studies that indicate the presence of active nitrogenase in these cultures [27]. We did not observe a significant up-regulation of this pathway in O_2_lim cell cultures (Additional file 1: Figures S14 and S15). Moreover, genes involved in two out of three *pmoCAB* operons that encode for three subunits of pMMO were differentially expressed in N_2_fix and O_2_lim cell cultures (Additional file 1: Figure S15). In both cultures, genes in *pmoCAB1* operon showed a significantly higher expression levels whereas those involved in *pmoCAB2* showed a strong decline in their expression as compared to μ_max_ cell cultures (Additional file 1: Tables S5 and S10). The expression of genes involved in tricarboxylic acid (TCA) cycle, carbon energy storage, carbon fixation, glycogen metabolism, and Calvin Benson Bassham (CBB) cycle pathways were either unchanged or showed a significant decline in N_2_fix and O_2_lim cell cultures as compared to μ_max_ cell cultures (Additional file 1: Tables S4, S6, S7, S10-S12).

### Base modifications and associated motifs

**Figure 3:**
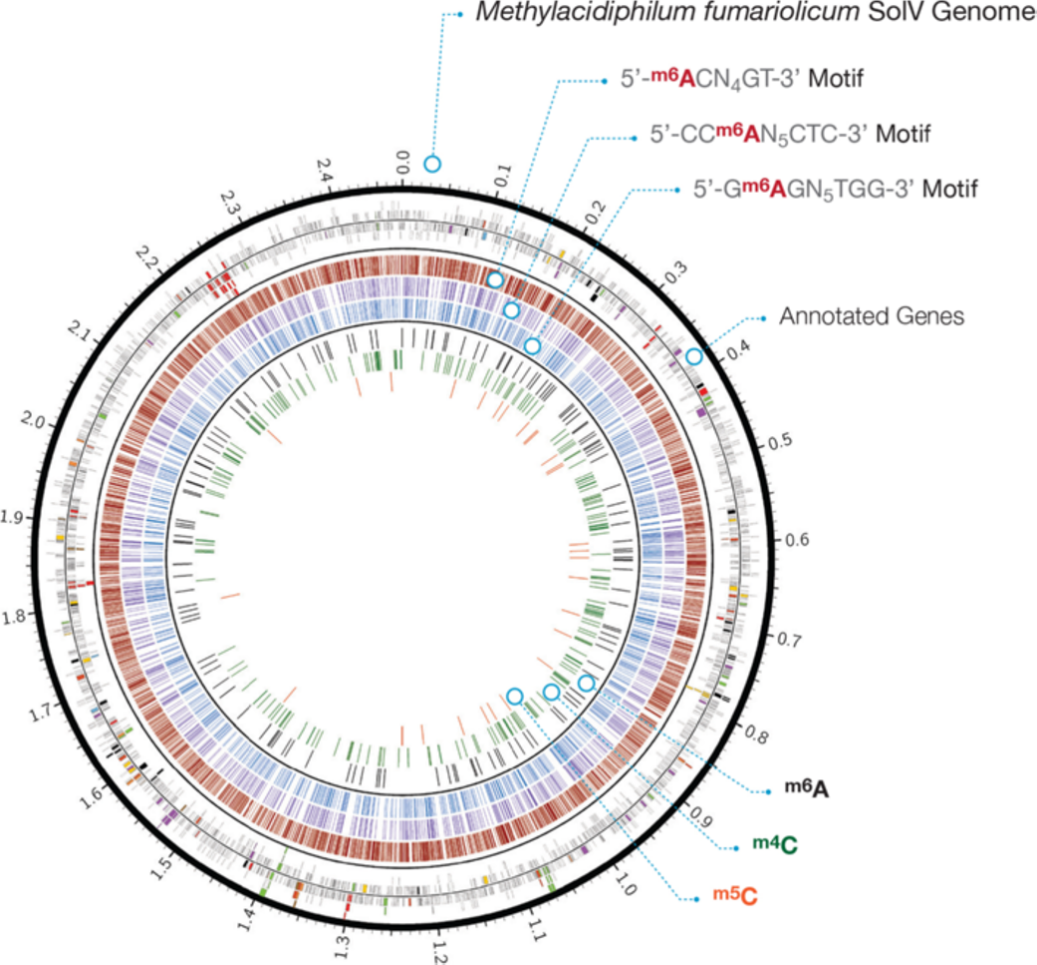
**The** ***Methylacidiphilum fumariolicum*** **SolV global methylation state.** The first inner circle shows the annotated genes and highlights those that are involved in key metabolic pathways. The second ring depicts methylated adenines that are associated with specific motifs. The placement of 5′-^**m6**^ **A**CN_4_GT-3′, 5′-CC^**m6**^ **A**N_5_CTC-3′, and 5′-G^**m6**^ **A**GN_5_TGG-3′ motifs are highlighted in red, purple, and blue ticks, respectively. Methylated bases that are not associated with any motifs are presented in the three innermost circles. The position of additional methylated adenines is shown in black. The position of m4C and m5C bases is marked in green and orange, respectively.

SMRT sequencing provides a unique platform for detecting N6-methyladenine (6mA), 4-methylcytosine (4mC), and 5-methylcytosine (5mC) bases across the genome [14]. The *M. fumariolicum* SolV genome contains multiple methyltransferases (Additional file 1: Table S13) and it should therefore be possible to detect different types of methylation. We have identified 16 different methyltransferases of which 12 could also be found in *M. infernorum* V4 with an average 59.6% identity (Additional file 1: Table S13). In addition, we could also find 7 RNA-methyltransferases, all of which were also present in *M. infernorum* V4 (70% identity). In order to assess the genome-wide methylation profile of *M. fumariolicum* SolV and identify the associated motifs, we performed two SMRT sequencing runs on an independently isolated and prepared sequencing library. To obtain a reliable polymerase kinetic signal for 5mC, the DNA was treated with Tet1 oxidation before sequencing [28]. This resulted in ~184,000 single-molecule sequencing reads (1,499 bp) with an average quality of 0.844 (Additional file 1: Figure S16) that yield to 287.4× average coverage of the reference genome. Sequencing reads were distributed normally across the genome with no missing bases and a consensus accuracy of 99.9998% (Additional file 1: Figure S17). Genome-wide analysis of polymerase kinetic profiles during SMRT sequencing enabled the identification of methylated adenine and cytosine bases. Adenine bases showed a very strong modification signal that strongly correlated with depth of coverage on each strand (Additional file 1: Figure S18). Based on the distribution of modification quality values (QV), a threshold for modification QV was increased to 50 to limit the amount of false positive modification calls (Additional file 1: Figure S19). We identified 8,588 6mAs, 220 4mCs, and 29 5mCs that were distributed across the entire genome (Figure 3). Whereas no motif was associated with cytosine methylation, sequence context analysis of methylated adenines indicated that 8,463 of methylated adenines (98.6%) were associated with three putative adenine methyltransferase recognition motifs: 5′-^6m^ACN_4_GT-3′ (6,151), 5′-CC^6m^AN_5_CTC-3′ (1,153), and 5′-G^6m^AGN_5_TGG-3′ (1,159) motifs (Figure 3; Additional file 1: Figure S20). 5′-CC^6m^AN_5_CTC-3′ and 5′-G^6m^AGN_5_TGG-3′ are partner motifs as they are reverse complement of each other. Adenine methylation was observed for over 98% of associated motifs in the genome (Table 3). Overall, 86.2% of methylated adenines and 84.3% of cytosine methylations reside in coding regions of the *M. fumariolicum* SolV genome.

**Table 3:** **Adenine motif statistics**

Motif ^1^	# motifs in genome	# motifs detected	% motifs detected	% intergenic	Mean coverage
G**A**GN_5_TGG	1,182	1,159	98.1	10.1	142.4
CC**A**N_5_CTC	1,182	1,153	97.6	9.7	143.1
**A**CN_4_GT	6,202	6,151	99.2	15.2	140.4

Motifs with a modification quality value >50 are considered.

^1^Methylated adenines are typed in bold.

## Discussion

It is essential to decipher a complete genetic makeup of *Methylacidiphilum fumariolicum* SolV to fully understand the underlying mechanisms used to oxidize methane in the hostile environmental conditions of volcanic and geothermal areas [4, 29]. Although the high-quality draft genome of *M. fumariolicum* SolV was previously published [6], efforts in finishing the genome remained unsuccessful due to the inherent limitations of second-generation sequencing technologies in resolving repeats and regions with extreme GC content. Here, we present the complete reference sequence of the *M. fumariolicum* SolV genome obtained using the single-molecule real-time (SMRT) sequencing strategy. SMRT sequencing of two large-insert template libraries followed by correction of sequencing errors resulted in high-quality *de novo* assembly of a single chromosome that is 2.48 Mbp in size, with a GC content of 41.5%. Due to the presence of long repeats and extremely low sequencing depth of Illumina GAII in GC-rich regions, this could not be achieved by combining short second-generation sequencing reads and long reads generated by SMRT sequencing [15, 30]. The *de novo* assembled complete genome of *M. fumariolicum* SolV has a very high quality as it holds a consensus accuracy of 99.9998% with single-molecule reads that were generated in two independent SMRT sequencing runs. We identified a number of misassemblies in the draft genome that were mainly the result of repeats and large fluctuation in Illumina GAII coverage. Despite recent advancements in chemistry and library preparation protocols of second-generation sequencing platforms, achieving a sufficient and uniform coverage on genomic regions with extreme GC content is challenging [31].

Genome annotation revealed the presence of 2,741 protein-encoding genes, 49 RNAs, and 314 functional subsystems. Moreover, the annotation of the complete genome sequence enabled the discovery of 458 genes that were previously missed in the draft genome. Closer analysis of newly annotated genes revealed the presence of two genes that belong to the C-subunit of pMMO. These genes were missed in the draft assembly. For new genes that fall in gaps or misassembled regions of the draft genome, only a minor fraction (11.2%) could be associated with functional subsystems. Other major pathways that could be associated with multiple genes were Ton and Tol transport systems and ribonucleotide reduction. After manually curating key metabolic pathways, a full Calvin-Benson-Bassham cycle was identified for carbon fixation whereas both the ribulose monophosphate and serine cycle pathways were absent. This is in concordance with our previous physiological studies [32]. The phylogenetic analysis of *pmoA* genes confirmed the separation of three species within the genus of *Methylacidiphilum* from other known methanotrophs. Moreover, 24.4% of annotated genes were highly conserved (>80% homology) between *M. fumariolicum* SolV and *M. infernorum* V4, whereas almost a third remained exclusive to the genome of *M. fumariolicum* SolV. The genome-wide analysis of expression profiles revealed a substantial down-regulation of genes in cell cultures with nitrogen fixation (N_2_fix) or oxygen limitation (O_2_lim) growth conditions. Except for genes that were originally missed or misassembled in the draft genome, our results are in full concordance to our previous transcriptome analysis [8] that was performed on the draft genome. The expression of genes involved in TCA cycle, carbon energy storage, carbon fixation, glycogen metabolism, and CBB cycle pathways were either unchanged or declined. The prominent down-regulation of genes is expected as less energy production is needed for these cell cultures given that they have been cultured at (4 times) reduced growth rates compared to μ_max_ cells. Furthermore, oxygen concentration may play a role in regulating pmoCAB operons as the expression of the *pmoCAB1* and *pmoCAB2* genes were significantly different in N_2_fix and O_2_lim cell cultures. It has also been shown that pMMO is differentially expressed under different growth conditions in *M. kamchatkense* [33]. The genome of species in the *Methylacidiphilum* genus includes all necessary genes to fix nitrogen [4, 7]. In the absence of ammonium and nitrate, genes that are involved in nitrogen fixation were significantly upregulated, which is in concordance with physiological studies that indicate the presence of active nitrogenase in N_2_fix cell cultures [27]. These observations are in agreement with the result of our previous transcriptome analysis that was performed on the draft genome [8].

DNA methylation is involved in a variety of biological processes and can have a profound physiological and functional consequence [13]. Despite its importance, the global DNA methylation state for most of the bacterial kingdom is poorly understood. The genome of *M. fumariolicum* SolV consist of several methyltransferases, including three polypeptides of Type I restriction-modification (RM) system. The absence of these genes in the genome of *M. infernorum* V4 suggests that methylation process can be regulated differently between species in the *Methylacidiphilum* genus. Comparative analysis of methylation patterns between these bacteria can be performed to elucidate the underlying mechanisms through which the genome integrity, gene regulation and defence processes are maintained given that such data is available in the future. Here, we characterize the methylation state of the *M. fumariolicum* SolV genome at a base-pair resolution by performing two SMRT sequencing runs on a single, Tet1 treated library. The result indicates a genome-wide adenine methylation that is associated with 5′-^**m6**^**A**CN_4_GT-3′, 5′-CC^**m6**^**A**N_5_CTC-3′, and 5′-G^**m6**^**A**GN_5_TGG-3′ motifs. Of 8,566 motif sites in the genome, only 103 sites were considered unmethylated under our growth condition. Although we were able to identify 220 4mCs and 29 5mCs, cytosine methylations were not associated with any specific motifs. To our knowledge, both 6mA motifs are potentially novel. It is possible that *M. fumariolicum* SolV contains genomic regions that are actively evolved against any occurrence of the 5′-CC^**m6**^**A**N_5_CTC-3′ system as there are several regions without any methylation of this kind. Mobile elements can, in principle, contribute to avoiding the RM systems. However, our analysis rules out their involvement since none were identified in the *M. fumariolicum* SolV genome (data not shown). Further studies are needed to reveal the underlying mechanisms for the negative selection of this methylation motif in this bacterium. In addition, it is essential to investigate the global influence of identified methylation motifs on gene expression that can be differentially regulated throughout the cell cycle and their affect on the physiology and function of this bacterium. This study provides a comprehensive atlas of *M. fumariolicum* SolV genome that allows for further transcriptome and epigenetic analysis of cell cultures under different growth conditions and stage of cell cycle to unravel the mechanisms through which methane oxidation is regulated in harsh fumarolic conditions.

## Conclusions

In this study, we reveal the complete genome sequence of *Methylacidiphilum fumariolicum* strain SolV and performed a thorough analysis of its genetic makeup, using a single-molecule real-time sequencing strategy. The finished sequence of a single chromosome enabled us to provide insights on genes that were missed due to gaps in the draft genome that were mainly caused by the limitations of second-generation sequencing technologies, owing to repetitiveness and high GC content of these regions. In addition, the complete genome sequence allowed us to expose misassemblies and perform a comparative analysis between genomes of the *M. fumariolicum* SolV and *M. infernorum* V4. For the first time, we provide a high-resolution and global methylation state of a *Methylacidiphilum* bacterium and the associated motifs at a base-pair resolution. Unravelling the *M. fumariolicum* SolV genome and its epigenetic regulation allow for robust characterization of biological processes that are involved in oxidizing methane. In turn, they offer a better understanding of the evolution, the underlying physiological and ecological properties of SolV and other *Methylacidiphilum* strains.

## Methods

### Bacterial growth conditions

*Methylacidiphilum fumariolicum* SolV used in this study was originally isolated from the volcanic re-gion Campi Flegrei, in Italy near Naples [3]. The cells were grown in medium containing g l-1: MgCl2.6H2O, 0.08; CaHPO_4_.2H_2_O, 0.44; Na_2_SO_4_, 0.14; K_2_SO_4_, 0.35; (NH_4_)_2_SO_4_, 0.26; 1 ml l^-1^ trace element solution [27] and 2% (v/v) autoclaved fangaia soil extract (liquid obtained from the Fangai mud pool at Pozzuoli in Italy). The pH and temperature for growth were 2 and 55°C, respectively. To obtain DNA for *de novo* assembly and modification analysis, cells were grown with methane at μ_max_ conditions as described in [3].

### Genomic DNA preparation

Genomic DNA of *Methylacidiphilum fumariolicum* SolV was isolated from cultures using the cetyltrimethylammonium bromide (CTAB) method described before [34] but without the use of lytic enzymes.

### Sequencing

SMRTbell DNA template libraries were prepared according to the manufacturer’s specification after the fragmentation with G-tubes (Covaris). Two different SMRTbell template library sizes were used, which had an average insert size of ~5 Kb and ~20 Kb. Subsequently, fragmented DNA was end-repaired and ligated to hairpin adapters. SMRT sequencing was carried out on the Pacific Biosciences RS according to standard protocols, 4 SMRT cells with the C1 chemistry (diffusion loading, 1 × 90 min, 5 Kb fragment size) and 6 SMRT cells with the XL binding kit used in conjunction with the C2 sequencing kit (Magbead loading, 1 × 120 min, 20 Kb fragment size). To enhance the 5mC kinetics analysis, 5mC residues were converted to 5caC using the 5mC Tet1 Oxidation kit (WiseGene), according to the manufacturer’s specification. Subsequently, for the purpose of base modification analysis, SMRTbell template libraries (insert size of ~2 Kb) were prepared and sequenced on two additional SMRT cells as described above. All runs were processed using the standard primary data analysis.

### De novo genome assembly

All continuous long reads from the first eight SMRT sequencing runs that were longer than 300 bp and passed the quality threshold of 0.75 were merged. Subsequently, the hierarchical genome-assembly process (HGAP) pipeline [16] was used to correct the sequencing errors by setting the seed length to 1500 bp for constructing preassemblies. The resulting corrected and preassembled reads were fed into the revised version of the Celera Assembler [15, 35] that is most suited for long corrected PacBio reads, owing to the use of the overlap-layout-consensus (OLC) strategy. Since SMRT sequencing shows very little variations of the quality throughout the reads [15], no quality values were used during the assembly. In addition, the BOGART unitigger and the default parameters (except the *mersize* of 14) were used. To validate the quality of the assembly and determine the final genome sequence, Quiver consensus algorithm [16] and reads from two additional SMRT sequencing runs were used. Quiver takes advantage of the full information from the raw pulse and base-calls that are generated during the SMRT sequencing to infer the best consensus sequence [16]. To further evaluate the quality of the final genome assembly, the consensus sequence were circularised and compared to the high quality draft genome [6] that was previously generated using Illumina and Roche 454 reads. Sequence gaps in the draft genome were further characterized using BLAST [36] and a custom Python script. Additional data analyses were carried out in R and Matlab. Gepard [37] and Circos [38] were used for visualization.

### Annotations

The origin and terminus of DNA replication were determined after GC Skew and cumulative GC Skew analysis performed by GenSkew (http://genskew.csb.univie.ac.at). Annotation of the assembled genome was performed using RAST prokaryotic genome annotation service [18]. The previously annotated draft genome of *M. fumariolicum* SolV [3, 6] and the complete genome of *M. infernorum* V4 [7] were used to aid the identification of coding and functional noncoding sequences. Genomic repeats and other structural variations were identified using Nucmer [39] and filtered according to length threshold of 500 bp and 95% copy identity. Tandem repeats were separately identified using Tandem Repeat Finder online service [40].

### Phylogenetic analysis

For the phylogenetic analysis of *M. fumariolicum* SolV, we used the *pmoA* gene that is commonly used as a phylogenetic marker for methanotrophic bacteria. The complete sequence of the *pmoA* and *amoA* genes were analysed as previously described [3, 4, 41]. Phylogeny.fr analysis pipeline [42] was used to construct the phylogeny. DNA and protein sequences were aligned using ProbCons [43] and were subsequently curated using Gblocks [44]. MEGA 5 [45] and PhyML [46] were used to determine the best fitting model and to construct the phylogenetic tree based on the maximum likelihood strategy. For validation purposes, the trees were bootstrapped for 100 times.

### Comparative genome analysis

The final genome sequence of *M. fumariolicum* SolV was compared to the genome sequence of a closely related bacterial strain, *M. infernorum* V4. BLAST [36] and Nucmer [39] were used to highlight sequence similarities between the two strains. In addition, we used RAST [18] to compare the conservation of annotated genes and pathways.

### Transcriptome analysis

The Illumina RNA-Seq data from cell cultures at maximum growth rate (μ_max_; GSM995700), cell cultures in a Nitrogen fixing environment (N_2_fix; GSM995701), and cell cultures in an Oxygen deprived environment (O_2_lim; GSM995702) were separately aligned to the complete genome sequence and analysed using Generic Transcriptome Analysis Pipeline (GENTRAP; https://git.lumc.nl/rig-framework/gentrap/tree/master). edgeR [47] was used to identify genes that show significantly variable expression profiles under different conditions. Additional analysis was carried out in R and Matlab.

### Base modification analysis

After aligning sequencing reads to the assembled genome, kinetic signals acquired during Pacific Biosciences SMRT sequencing were processed for all genomic positions using a previously described protocol [14, 48]. To identify modified bases, Pacific Biosciences SMRT Portal analysis platform 2.0 was used. SMRT Portal uses an *in silico* kinetic model and a t-test based scoring system to detect modified bases. In order to accurately identify methylated bases, a threshold of 50 for log-transformed *P* value was used. The threshold was optimized according to the distribution of *P* values for different bases, minimizing the false positive rate. The identification of sequence motifs was performed using the SMRT Portal. Additional data analysis was carried out in R using Pacific Biosciences R-Kinetics package, available at https://github.com/PacificBiosciences/R-kinetics.

### Data availability

The aligned whole-genome shotgun sequencing reads, RNA-Seq data, and complete genome sequence of *Methylacidiphilum fumariolicum* SolV are deposited at the European Nucleotide Archive under the study accession number PRJEB6910 (http://www.ebi.ac.uk/ena/data/view/PRJEB6910).

## Electronic supplementary material

Additional file 1: **Supplementary Figures and Tables.** (PDF 17 MB)

## References

[1] Dunfield PF, Yuryev A, Senin P, Smirnova AV, Stott MB, Hou S, Ly B, Saw JH, Zhou Z, Ren Y, Wang J, Mountain BW, Crowe MA, Weatherby TM, Bodelier PL, Liesack W, Feng L, Wang L, Alam M (2007). Methane oxidation by an extremely acidophilic bacterium of the phylum Verrucomicrobia. Nature.

[2] Islam T, Jensen S, Reigstad LJ, Larsen O, Birkeland NK (2008). Methane oxidation at 55 degrees C and pH 2 by a thermoacidophilic bacterium belonging to the Verrucomicrobia phylum. Proc Natl Acad Sci U S A.

[3] Pol A, Heijmans K, Harhangi HR, Tedesco D, Jetten MS, Op den Camp HJM (2007). Methanotrophy below pH 1 by a new Verrucomicrobia species. Nature.

[4] Op den Camp HJM, Islam T, Stott MB, Harhangi HR, Hynes A, Schouten S, Jetten MS, Birkeland NK, Pol A, Dunfield PF (2009). Environmental, genomic and taxonomic perspectives on methanotrophic Verrucomicrobia. Environm Microbiol Rep.

[5] Hanson RS, Hanson TE (1996). Methanotrophic bacteria. Microbiol Rev.

[6] Khadem AF, Wieczorek AS, Pol A, Vuilleumier S, Harhangi HR, Dunfield PF, Kalyuzhnaya MG, Murrell JC, Francoijs KJ, Stunnenberg HG, Stein LY, DiSpirito AA, Semrau JD, Lajus A, Medigue C, Klotz MG, Jetten MS, Op den Camp HJM (2012). Draft genome sequence of the volcano-inhabiting thermoacidophilic methanotroph *Methylacidiphilum fumariolicum* strain SolV. J Bacteriol.

[7] Hou S, Makarova KS, Saw JH, Senin P, Ly BV, Zhou Z, Ren Y, Wang J, Galperin MY, Omelchenko MV, Wolf YI, Yutin N, Koonin EV, Stott MB, Mountain BW, Crowe MA, Smirnova AV, Dunfield PF, Feng L, Wang L, Alam M (2008). Complete genome sequence of the extremely acidophilic methanotroph isolate V4, *Methylacidiphilum infernorum*, a representative of the bacterial phylum Verrucomicrobia. Biol Direct.

[8] Khadem AF, Pol A, Wieczorek AS, Jetten MS, Op den Camp HJM (2012). Metabolic Regulation of "Ca. Methylacidiphilum Fumariolicum" SolV Cells Grown Under Different Nitrogen and Oxygen Limitations. Front Microbiol.

[9] Gonzalez D, Kozdon JB, McAdams HH, Shapiro L, Collier J: **The functions of DNA methylation by CcrM in Caulobacter crescentus: a global approach.** *Nucleic Acids Res* 2014.,**42**(6)**:** 10.1093/nar/gkt1352 PMC3973325 24398711

[10] Wion D, Casadesus J (2006). N6-methyl-adenine: an epigenetic signal for DNA-protein interactions. Nat Rev Microbiol.

[11] Roberts RJ, Vincze T, Posfai J, Macelis D (2010). REBASE–a database for DNA restriction and modification: enzymes, genes and genomes. Nucleic Acids Res.

[12] Jeltsch A (2003). Maintenance of species identity and controlling speciation of bacteria: a new function for restriction/modification systems?. Gene.

[13] Davis BM, Chao MC, Waldor MK (2013). Entering the era of bacterial epigenomics with single molecule real time DNA sequencing. Curr Opin Microbiol.

[14] Flusberg BA, Webster DR, Lee JH, Travers KJ, Olivares EC, Clark TA, Korlach J, Turner SW (2010). Direct detection of DNA methylation during single-molecule, real-time sequencing. Nat Methods.

[15] Koren S, Schatz MC, Walenz BP, Martin J, Howard JT, Ganapathy G, Wang Z, Rasko DA, McCombie WR, Jarvis ED, Adam MP (2012). Hybrid error correction and de novo assembly of single-molecule sequencing reads. Nat Biotechnol.

[16] Chin CS, Alexander DH, Marks P, Klammer AA, Drake J, Heiner C, Clum A, Copeland A, Huddleston J, Eichler EE, Turner SW, Korlach J (2013). Nonhybrid, finished microbial genome assemblies from long-read SMRT sequencing data. Nat Methods.

[17] Au KF, Underwood JG, Lee L, Wong WH (2012). Improving PacBio long read accuracy by short read alignment. PloS One.

[18] Aziz RK, Bartels D, Best AA, DeJongh M, Disz T, Edwards RA, Formsma K, Gerdes S, Glass EM, Kubal M, Meyer F, Olsen GJ, Olson R, Osterman AL, Overbeek RA, McNeil LK, Paarmann D, Paczian T, Parrello B, Pusch GD, Reich C, Stevens R, Vassieva O, Vonstein V (2008). The RAST Server: rapid annotations using subsystems technology. BMC Genomics.

[19] Overbeek R, Begley T, Butler RM, Choudhuri JV, Chuang HY, Cohoon M, de Crecy-Lagard V, Diaz N, Disz T, Edwards R, Fonstein M, Frank ED, Gerdes S, Glass EM, Goesmann A, Hanson A, Iwata-Reuyl D, Jensen R, Jamshidi N, Krause L, Kubal M, Larsen N, Linke B, McHardy AC, Meyer F, Neuweger H, Olsen G, Olson R, Osterman A, Portnoy V (2005). The subsystems approach to genome annotation and its use in the project to annotate 1000 genomes. Nucleic Acids Res.

[20] Grigoriev A (1998). Analyzing genomes with cumulative skew diagrams. Nucleic Acids Res.

[21] Dedysh SN, Berestovskaya YY, Vasylieva LV, Belova SE, Khmelenina VN, Suzina NE, Trotsenko YA, Liesack W, Zavarzin GA (2004). Methylocella tundrae sp. nov., a novel methanotrophic bacterium from acidic tundra peatlands. Int J Syst Evol Microbiol.

[22] Vorobev AV, Baani M, Doronina NV, Brady AL, Liesack W, Dunfield PF, Dedysh SN (2011). Methyloferula stellata gen. nov., sp. nov., an acidophilic, obligately methanotrophic bacterium that possesses only a soluble methane monooxygenase. Int J Syst Evol Microbiol.

[23] Kolb S, Knief C, Stubner S, Conrad R (2003). Quantitative detection of methanotrophs in soil by novel pmoA-targeted real-time PCR assays. Appl Environ Microbiol.

[24] Holmes AJ, Costello A, Lidstrom ME, Murrell JC (1995). Evidence that particulate methane monooxygenase and ammonia monooxygenase may be evolutionarily related. FEMS Microbiol Lett.

[25] Luesken FA, Zhu B, Van Alen TA, Butler MK, Diaz MR, Song B, Op den Camp HJM, Jetten MS, Ettwig KF (2011). pmoA Primers for detection of anaerobic methanotrophs. Appl Environ Microbiol.

[26] McDonald IR, Murrell JC (1997). The particulate methane monooxygenase gene pmoA and its use as a functional gene probe for methanotrophs. FEMS Microbiol Lett.

[27] Khadem AF, Pol A, Jetten MS, Op den Camp HJM (2010). Nitrogen fixation by the verrucomicrobial methanotroph '*Methylacidiphilum fumariolicum*' SolV. Microbiology.

[28] Clark TA, Lu X, Luong K, Dai Q, Boitano M, Turner SW, He C, Korlach J (2013). Enhanced 5-methylcytosine detection in single-molecule, real-time sequencing via Tet1 oxidation. BMC Biol.

[29] Castaldi S, Tedesco D (2005). Methane production and consumption in an active volcanic environment of Southern Italy. Chemosphere.

[30] Ribeiro FJ, Przybylski D, Yin S, Sharpe T, Gnerre S, Abouelleil A, Berlin AM, Montmayeur A, Shea TP, Walker BJ, Young SK, Russ C, Nusbaum C, MacCallum I, Jaffe DB (2012). Finished bacterial genomes from shotgun sequence data. Genome Res.

[31] Quail MA, Smith M, Coupland P, Otto TD, Harris SR, Connor TR, Bertoni A, Swerdlow HP, Gu Y (2012). A tale of three next generation sequencing platforms: comparison of Ion Torrent, Pacific Biosciences and Illumina MiSeq sequencers. BMC Genomics.

[32] Khadem AF, Pol A, Wieczorek A, Mohammadi SS, Francoijs KJ, Stunnenberg HG, Jetten MS, Op den Camp HJM (2011). Autotrophic methanotrophy in verrucomicrobia: *Methylacidiphilum fumariolicum* SolV uses the calvin-benson-bassham cycle for carbon dioxide fixation. J Bacteriol.

[33] Erikstad HA, Jensen S, Keen TJ, Birkeland NK (2012). Differential expression of particulate methane monooxygenase genes in the verrucomicrobial methanotroph '*Methylacidiphilum kamchatkense*' Kam1. Extremophiles.

[34] Juretschko S, Timmermann G, Schmid M, Schleifer KH, Pommerening-Roser A, Koops HP, Wagner M (1998). Combined molecular and conventional analyses of nitrifying bacterium diversity in activated sludge: Nitrosococcus mobilis and Nitrospira-like bacteria as dominant populations. Appl Environ Microbiol.

[35] Myers EW, Sutton GG, Delcher AL, Dew IM, Fasulo DP, Flanigan MJ, Kravitz SA, Mobarry CM, Reinert KH, Remington KA, Anson EL, Bolanos RA, Chou HH, Jordan CM, Halpern AL, Lonardi S, Beasley EM, Brandon RC, Chen L, Dunn PJ, Lai Z, Liang Y, Nusskern DR, Zhan M, Zhang Q, Zheng X, Rubin GM, Adams MD, Venter JC (2000). A whole-genome assembly of Drosophila. Science (New York, NY).

[36] Altschul SF, Gish W, Miller W, Myers EW, Lipman DJ (1990). Basic local alignment search tool. J Mol Biol.

[37] Krumsiek J, Arnold R, Rattei T (2007). Gepard: a rapid and sensitive tool for creating dotplots on genome scale. Bioinformatics.

[38] Krzywinski M, Schein J, Birol I, Connors J, Gascoyne R, Horsman D, Jones SJ, Marra MA (2009). Circos: an information aesthetic for comparative genomics. Genome Res.

[39] Kurtz S, Phillippy A, Delcher AL, Smoot M, Shumway M, Antonescu C, Salzberg SL (2004). Versatile and open software for comparing large genomes. Genome Biol.

[40] Benson G (1999). Tandem repeats finder: a program to analyze DNA sequences. Nucleic Acids Res.

[41] Margulies M, Egholm M, Altman WE, Attiya S, Bader JS, Bemben LA, Berka J, Braverman MS, Chen YJ, Chen Z, Dewell SB, Du L, Fierro JM, Gomes XV, Godwin BC, He W, Helgesen S, Ho CH, Irzyk GP, Jando SC, Alenquer ML, Jarvie TP, Jirage KB, Kim JB, Knight JR, Lanza JR, Leamon JH, Lefkowitz SM, Lei M, Li J (2005). Genome sequencing in microfabricated high-density picolitre reactors. Nature.

[42] Dereeper A, Guignon V, Blanc G, Audic S, Buffet S, Chevenet F, Dufayard JF, Guindon S, Lefort V, Lescot M, Claverie JM, Gascuel O (2008). Phylogeny.fr: robust phylogenetic analysis for the non-specialist. Nucleic Acids Res.

[43] Do CB, Mahabhashyam MS, Brudno M, Batzoglou S (2005). ProbCons: Probabilistic consistency-based multiple sequence alignment. Genome Res.

[44] Talavera G, Castresana J (2007). Improvement of phylogenies after removing divergent and ambiguously aligned blocks from protein sequence alignments. Syst Biol.

[45] Tamura K, Peterson D, Peterson N, Stecher G, Nei M, Kumar S (2011). MEGA5: molecular evolutionary genetics analysis using maximum likelihood, evolutionary distance, and maximum parsimony methods. Mol Biol Evol.

[46] Guindon S, Dufayard JF, Lefort V, Anisimova M, Hordijk W, Gascuel O (2010). New algorithms and methods to estimate maximum-likelihood phylogenies: assessing the performance of PhyML 3.0. Syst Biol.

[47] Robinson MD, McCarthy DJ, Smyth GK (2010). edgeR: a Bioconductor package for differential expression analysis of digital gene expression data. Bioinformatics.

[48] Clark TA, Murray IA, Morgan RD, Kislyuk AO, Spittle KE, Boitano M, Fomenkov A, Roberts RJ, Korlach J (2012). Characterization of DNA methyltransferase specificities using single-molecule, real-time DNA sequencing. Nucleic Acids Res.

