# Supplementary material for: The genomic landscape of the verrucomicrobial methanotroph Methylacidiphilum fumariolicum SolV
Source: BMC Genomics. 2014 Oct 20;15(1):914. doi: 10.1186/1471-2164-15-914 (PMC4210602; doi:10.1186/1471-2164-15-914)
Supplement: Supplementary file 1 — Additional file 1: Supplementary Figures and Tables. (PDF 17 MB) [file 12864_2014_6596_MOESM1_ESM.pdf]

SUPPLEMENTARY MATERIALS

The genomic landscape of the verrucomicrobial methanotroph *Methylacidiphilum fumariolicum* SolV

Seyed Yahya Anvar[1,2,*], Jeroen Frank[2], Arjan Pol[3], Arnoud Schmitz[2], Ken Kraaijeveld[2,4], Johan T. den Dunnen[1,2], Huub J.M. Op den Camp[3,*]

[1] Department of Human Genetics and [2] Leiden Genome Technology Center, Leiden University Medical Center, Leiden, the Netherlands. [3] Department of Microbiology, Radboud University, Nijmegen, the Netherlands. [4] Department of Ecological Science, Section Animal Ecology, VU University Amsterdam, Amsterdam, the Netherlands.

∗ To whom correspondence should be addressed:

SYA	Tel: 0031715268559; Email: s.y.anvar@lumc.nl
HJMOdC	Tel: 0031243652657; Email: h.opdencamp@science.ru.nl

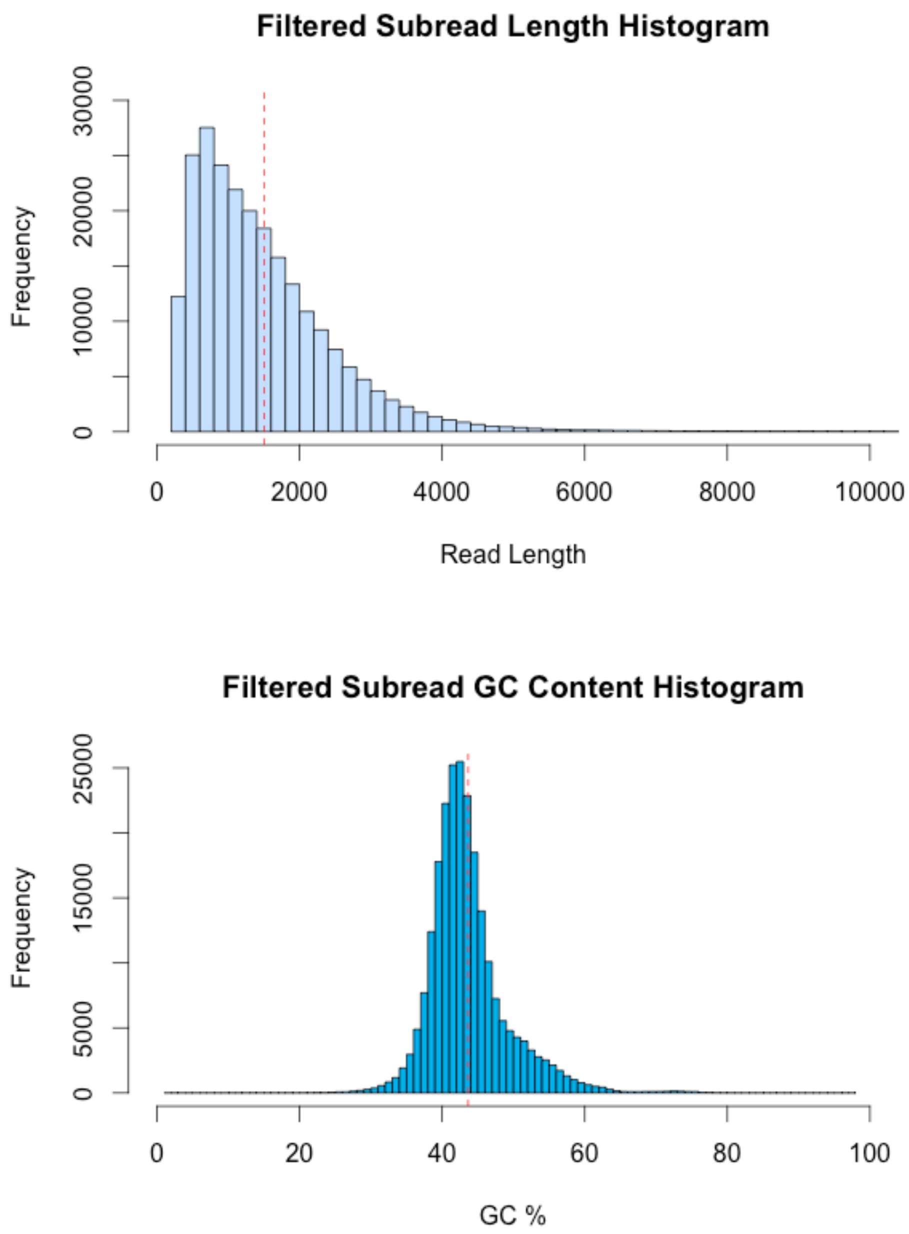

Supplementary Figure 1 – PacBio read-length and GC-content distributions prior to HGAP error correction. The red lines depict the average read-length and GC-content, respectively. GC-content is averaged per sequencing read. Plots are generated in R.

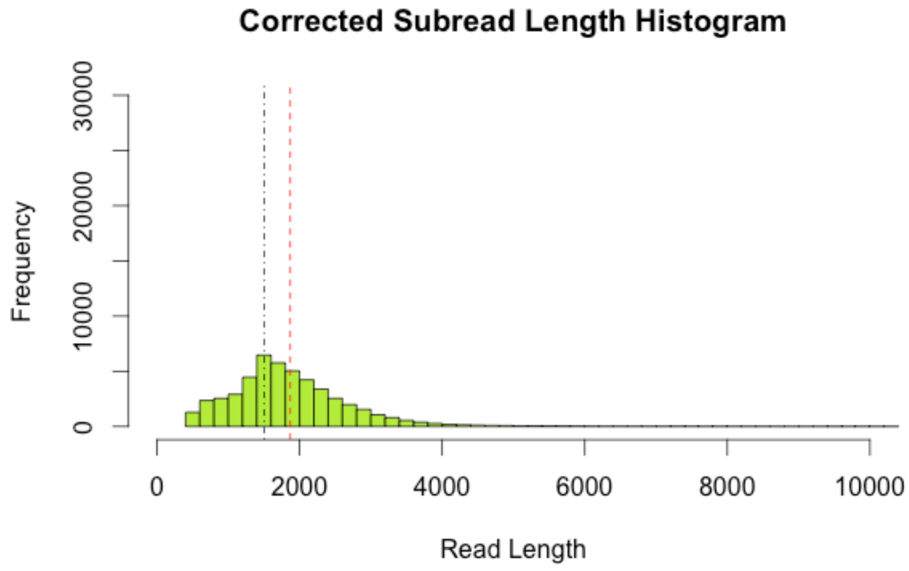

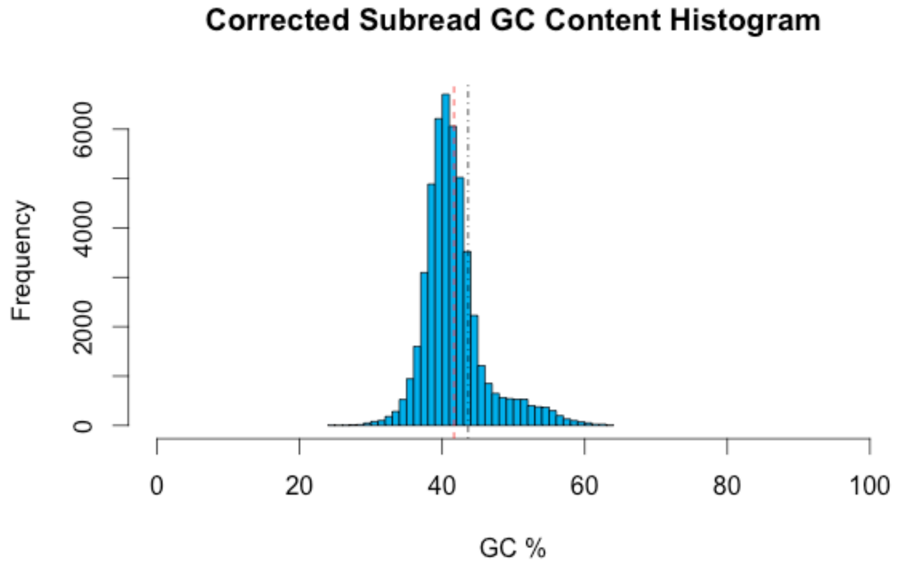

Supplementary Figure 2 – PacBio read-length and GC-content distributions after HGAP error correction. The red lines depict the average read-length and GC-content, respectively. The dark grey lines represent average read-length and GC-content prior to error correction. Plots are generated in R.

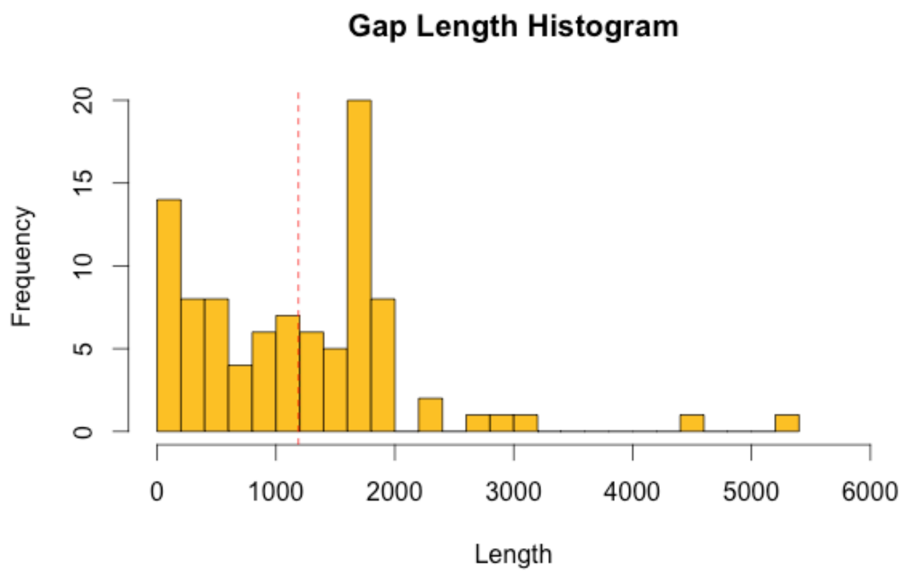

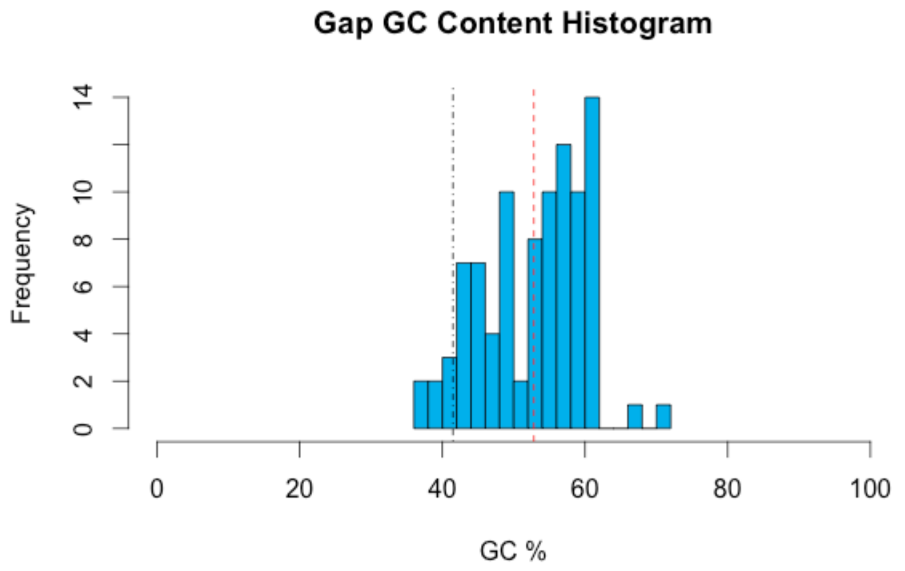

Supplementary Figure 3 – Length and GC content distributions of gaps in the draft genome compared to the final assembly. The red lines depict the average read length and GC content, respectively. The dark grey line represents the average GC content of the entire *Methylacidiphilum fumariolicum* SolV genome.

Supplementary Table 1 – Read statistics of Illumina GAII and Roche 454 datasets previously generated for *M. fumariolicum* SolV and were used during draft genome assembly.

	Illumina GAII	Roche 454
Number of reads	15,346,966	752,296
Total nucleotides	920,989,270	78,061,927
Mean read length	60bp	104bp
5th percentile	34bp	82bp
95th percentile	75bp	122bp
Maximum length	75bp	215bp
GC content	40.99%	41.93%
Coverage depth	369.88×	31.35×

Summary statistics are determined after trimming and filtering.

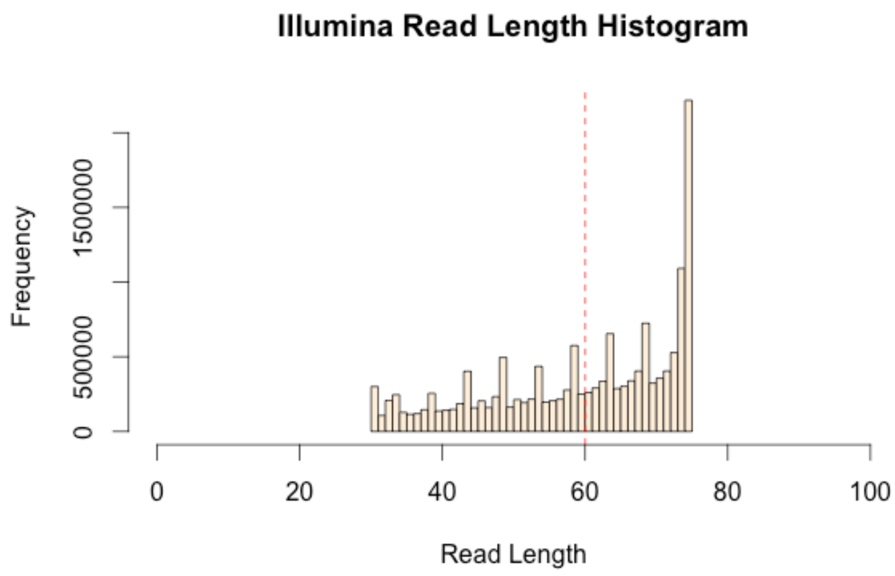

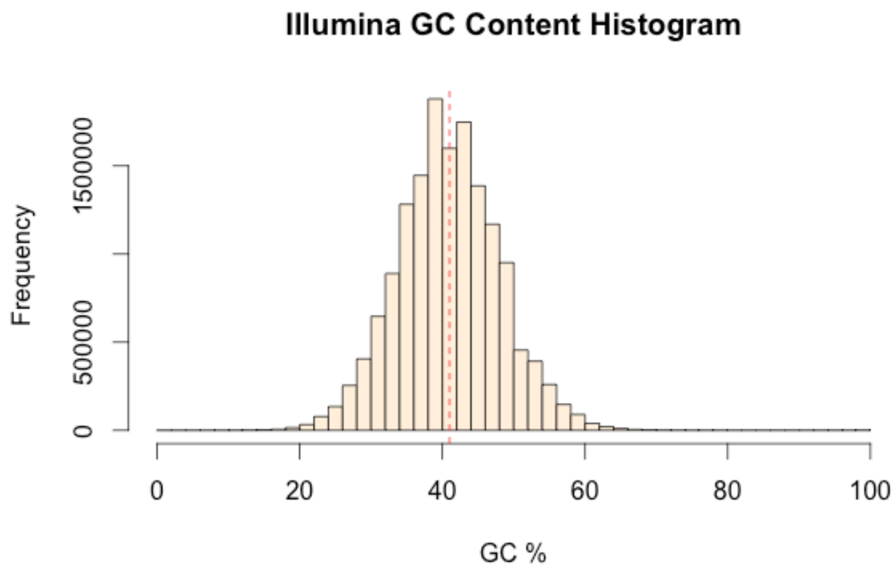

Supplementary Figure 4 – Illumina GAII read length and GC content distributions. The red lines depict the average read length and GC content, respectively.

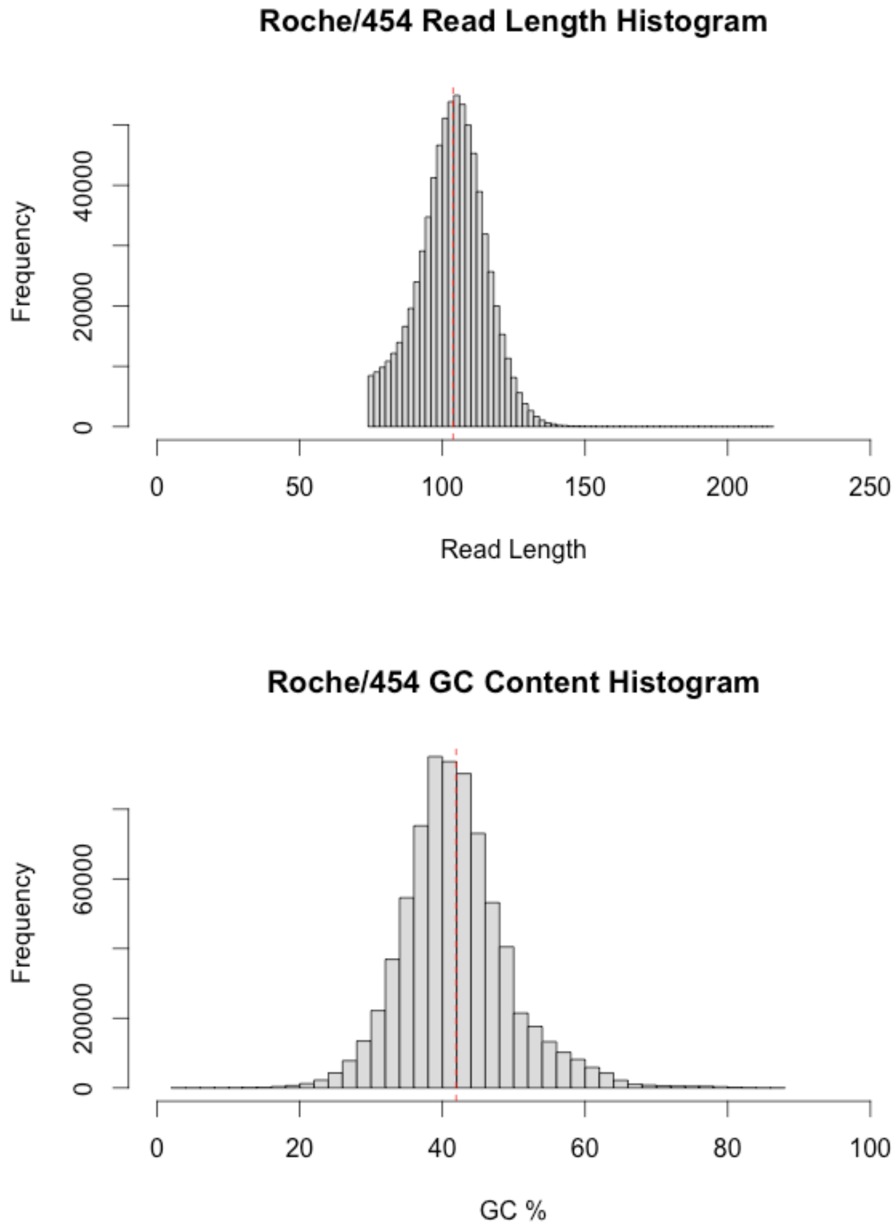

Supplementary Figure 5 – Roche 454 read length and GC content distributions. The red lines depict the average read length and GC content, respectively.

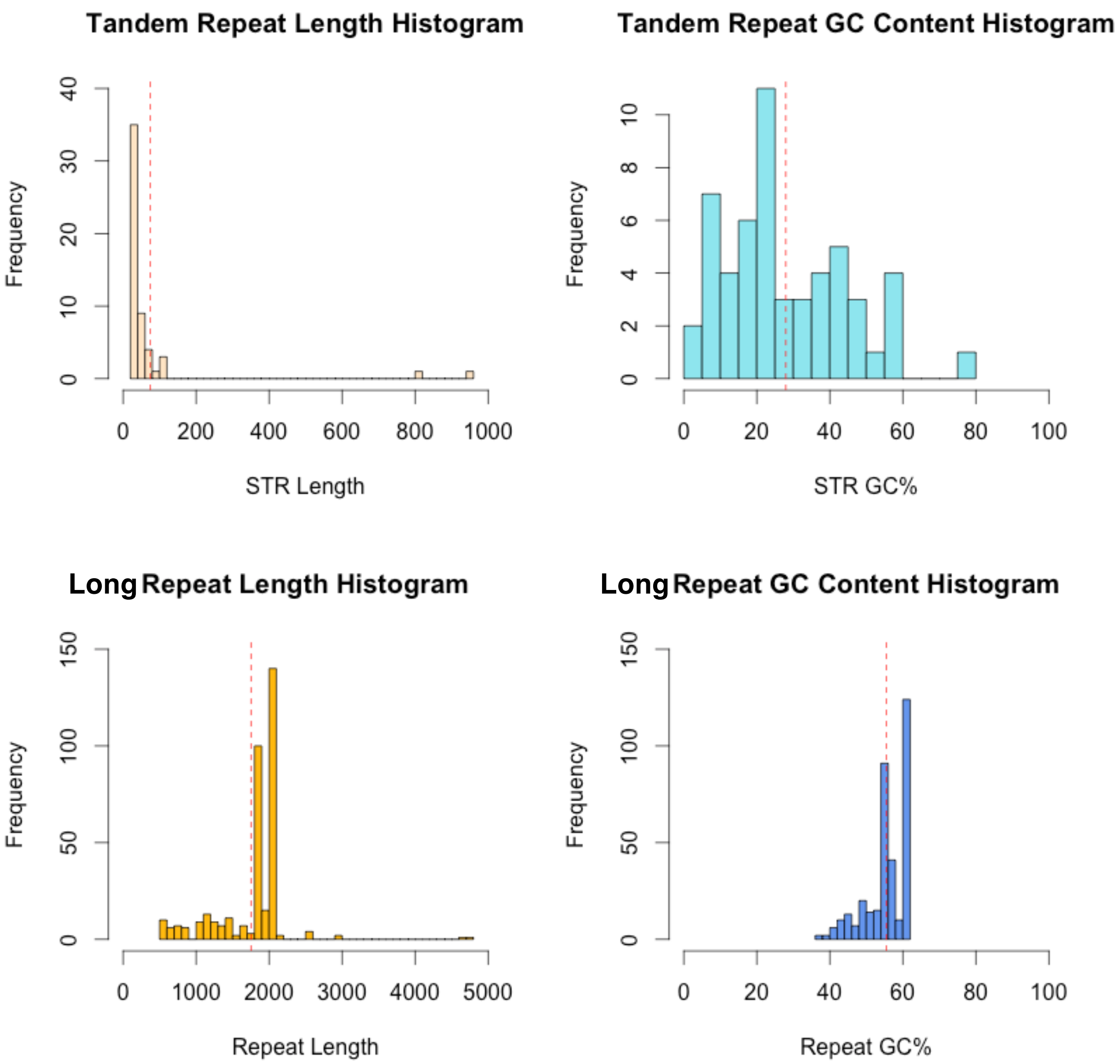

Supplementary Figure 6 – Length and GC content distributions of repeats across the entire genome. The top panels depict the distributions for tandem repeats whereas the bottom panels shows distributions for larger repeats (> 500 bp) that are not in tandem. The red lines depict the average read length and GC content, respectively.

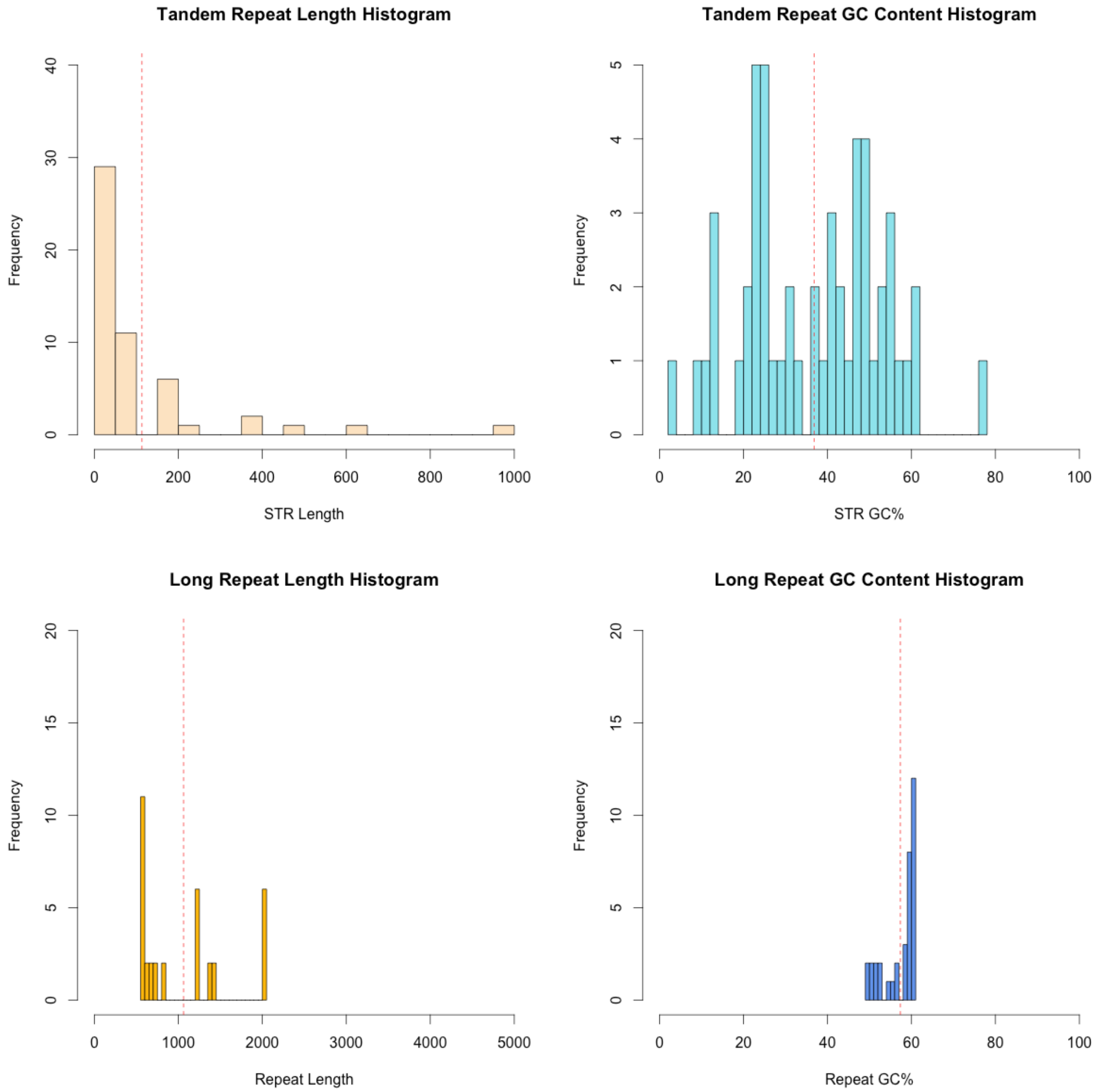

Supplementary Figure 7 – Length and GC content distributions of repeats across the genome of *M. infernorum* V4. The top panels depict the distributions for tandem repeats whereas the bottom panels shows distributions for larger repeats (> 500 bp) that are not in tandem. The red lines depict the average read length and GC content, respectively. There are 52 short tandems (47 bp) with average GC content of 37%. We also identified 35 long repeats (819 bp) with an average GC content of 57%.

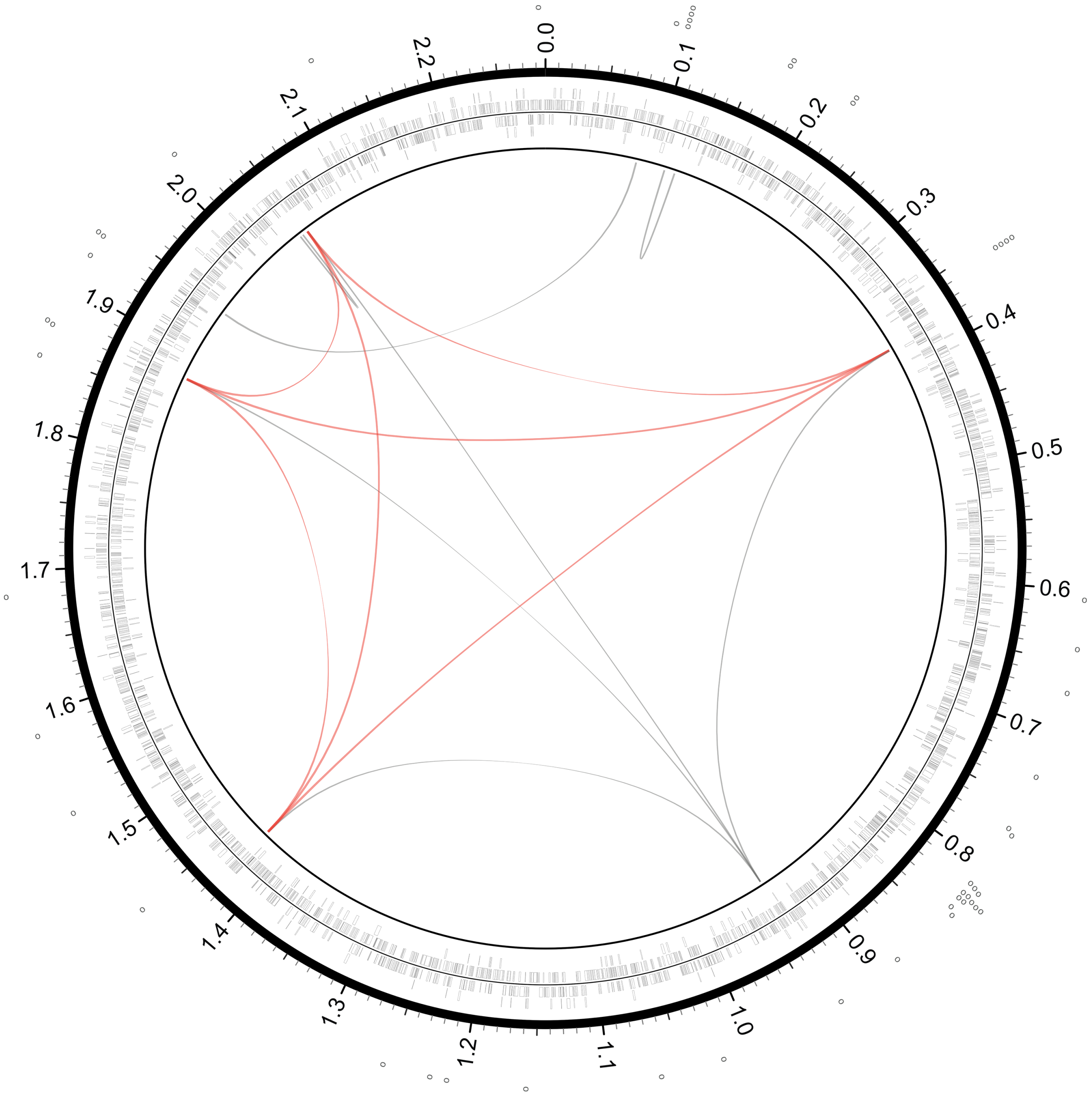

Supplementary Figure 8 – Short tandem and large repeats across the genome of *M. infernorum* V4. The circos plot illustrates the position of short tandem (outer ring) and large (>500 bp) repeats in the genome. Large repeats that are longer than 2,000 bp are depicted in red.

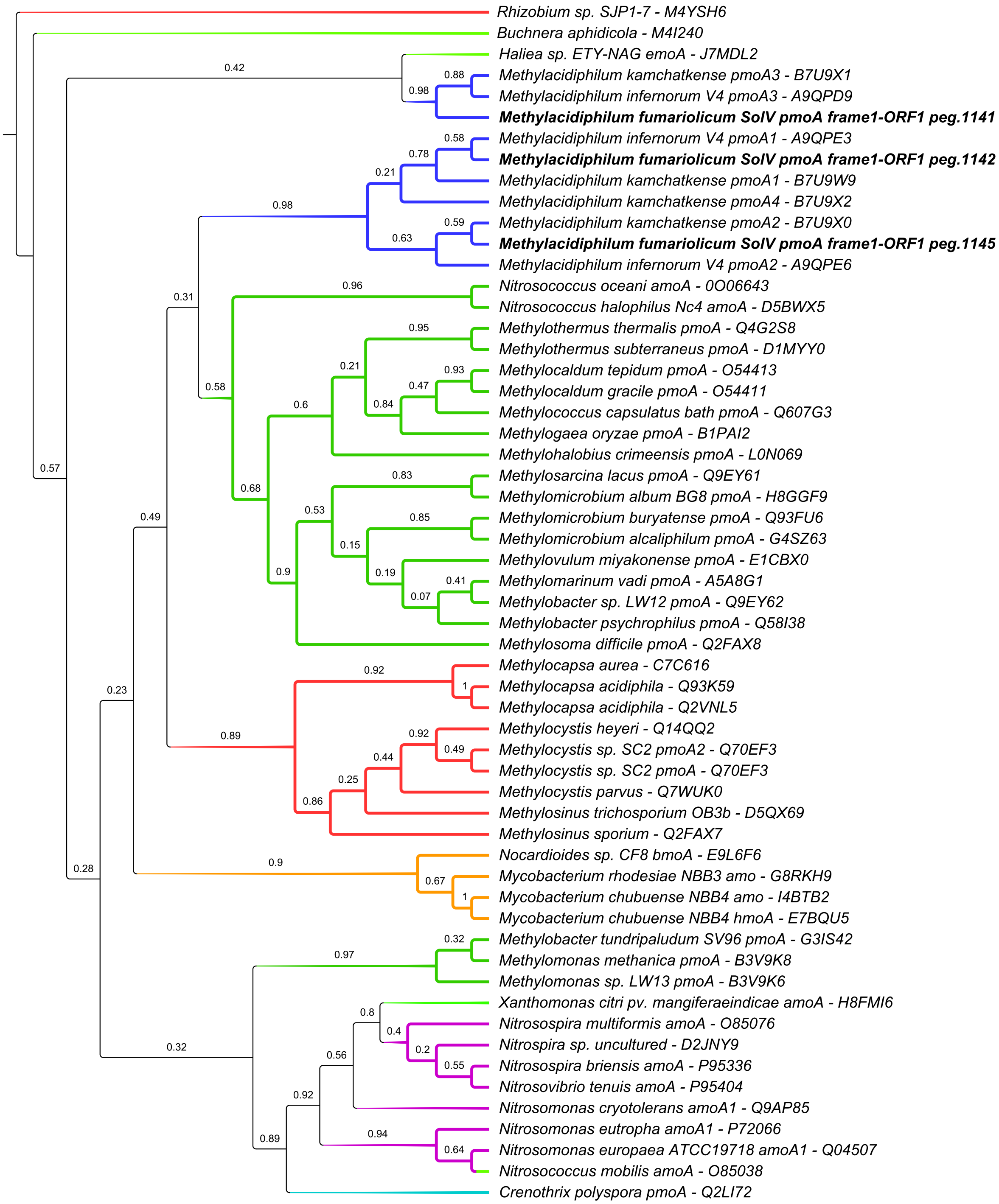

Supplementary Figure 9 – Phylogenetic relationships between annotated pmoA proteins. Colouring indicates different clusters within the constructed tree. The pmoA copies from the *Methylacidiphilum fumariolicum* SolV genome are indicated in bold. Main clusters are highlighted in colours. UniProt accession numbers are indicated for all sequences that are used in this analysis.

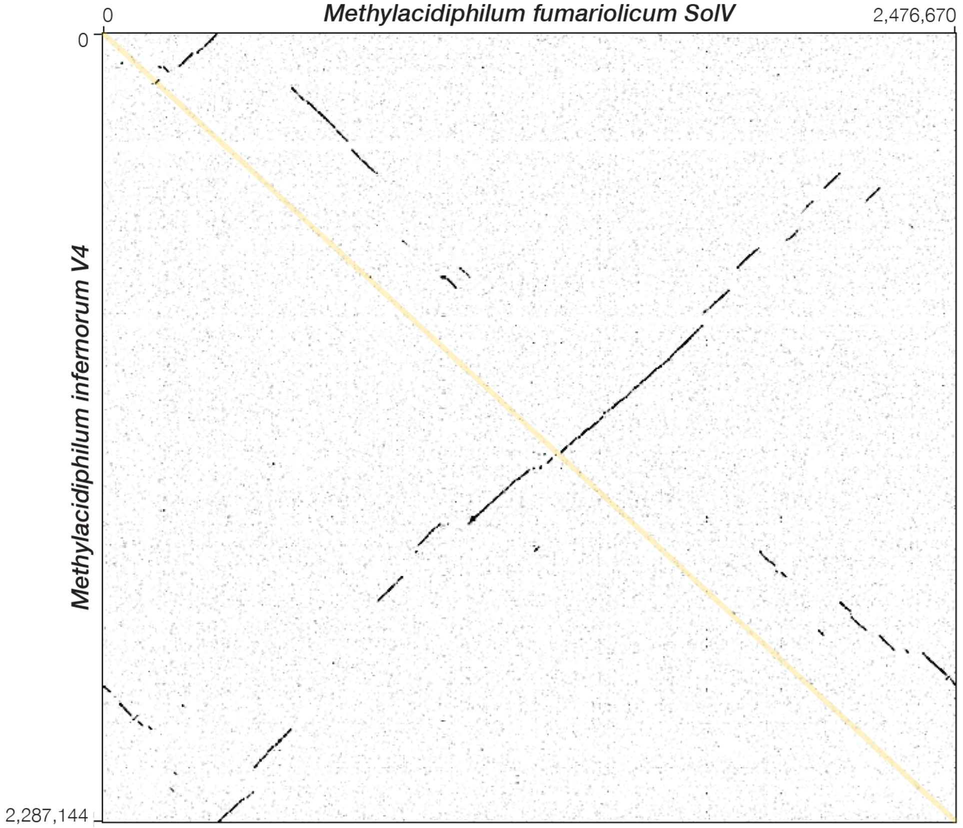

Supplementary Figure 10 – Dot plot of the *Methylacidiphilum fumariolicum* SolV assembly compared to the genome of *Methylacidiphilum infernorum* V4. The genomic sequence of the final assembly is aligned to the reference genome of *Methylacidiphilum infernorum* V4 [1] using Gepard [2]. Deviations from the yellow line indicate structural variations between two genomes.

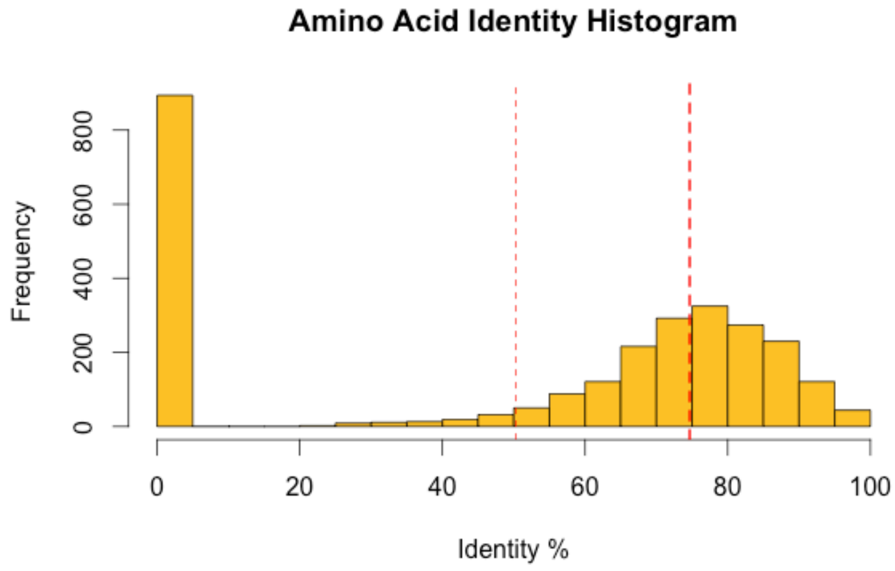

Supplementary Figure 11 – Amino acid identity plot between *Methylacidiphilum fumariolicum* SolV and *Methylacidiphilum infernorum* V4 genomes. The amino acid identities are calculated for all PEGs (protein encoding genes). Thin dash line depicts the overall average amino acid identity between two genomes and the thick dash line indicates the average for non-zero amino acid identities.

Supplementary Table 2 – A list of subsystems that are represented by genes that are located in gaps or misassembled regions of the draft genome of *Methylacidiphilum fumariolicum* SolV.

Subsystem	Number of Missed Genes Partially	Completely
Arginine and Ornithine Degradation, Glutamate dehydrogenases, Glutamate dehydrogenases, Aspartate and Asparagine Biosynthesis, Proline Synthesis	0	1
Carbon Starvation	0	1
Ribonuclease H, Ribonucleases in Bacillus	0	1
Chorismate: Intermediate for synthesis of Tryptophan, PAPA antibiotics, 3-hydroxyanthranilate	0	2
Cobalt-zinc-cadmium resistance	1	1
Group II intron-associated genes	0	1
Hopanes	0	1
Ton and Tol transport systems	1	3
Ammonia assimilation	1	0
DNA repair, bacterial	1	0
Iron-sulfur cluster assembly	1	0
Maltose and Maltodextrin Utilization, Trehalose Biosynthesis	1	0
Ribonucleotide reduction	3	0
NA	79	79
Total	88	90

Supplementary Table 3 – Summary statistics on conserved PEGs between *Methylacidiphilum fumariolicum* SolV and *Methylacidiphilum infernorum* V4.

	Total	Assigned to subsystems[1]
PEGs exclusive to SolV	894	101
Shared PEGs	1,847 *	753 **
> 50%	1,761	
> 60%	1,623	
> 70%	1,286	
> 80%	669	
> 90%	165	
PEGs exclusive to V4	718	46

[1] Summary statistics for PEGs that are assigned to subsystems are extracted from RAST annotations.

* All non-zero amino acid identities are considered.

** PEGs that are associated with subsystems have high amino acid identity between genomes.

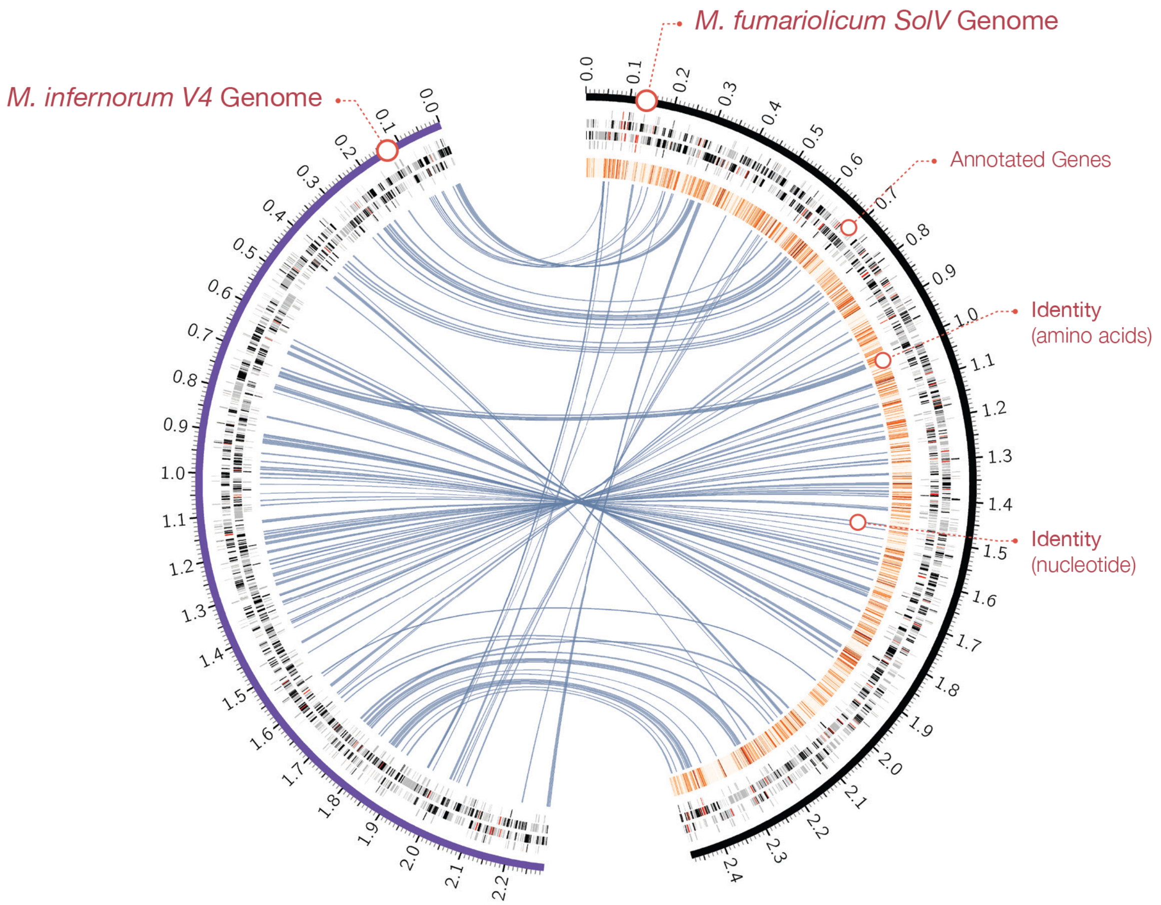

Supplementary Figure 12 – Circos plot of similarities between *Methylacidiphilum fumariolicum* SolV and *Methylacidiphilum infernorum* V4 [1] at the nucleotide and amino acid level. Genes that are conserved between two genomes are depicted in black. Genes that are exclusive to a single genome is highlighted in red. All other annotated genes are indicated in light grey. Regions with amino acid identities greater than 70% are indicated in orange. Links highlight conserved regions with at least 70% nucleotide identity.

Supplementary Table 4 – Summary statistics of gene expression for tricarboxylic acid (TCA) cycle pathway in *M. fumariolicum* SolV under maximum growth (μmax), nitrogen fixation (N_2fix) or oxygen limited (O_2lim) conditions.

		Genomic Position		μmax	N2 fixation		O2 limitation	
**PEGs**	**Function**	**Start**	**End**	**CPM***	**FC***	**FDR***	**FC***	**FDR***
mfum_765	polysaccharide deacetylase family protein	731750	732530	6.457	-1.534	2.59E-10	-0.031	1.00E+00
mfum_271	2-oxoglutarate dehydrogenase E1 component (EC 1.2.4.2)	248472	251241	8.311	-1.378	1.45E-09	-0.652	2.77E-02
mfum_1750	Aconitate hydratase (EC 4.2.1.3)	1590467	1593212	6.738	-1.272	1.10E-07	-1.520	1.76E-03
mfum_1786	Dihydrolipoamide dehydrogenase (EC 1.8.1.4)	1628692	1630078	6.237	-1.132	3.84E-06	-0.641	1.40E-02
mfum_448	Pyruvate kinase family protein	411114	412932	5.036	-1.130	2.86E-05	-1.112	5.37E-03
mfum_270	Dihydrolipoamide succinyltransferase component (E2) of 2-oxoglutarate dehydrogenase complex (EC 2.3.1.61)	247252	248419	6.758	-0.953	6.88E-05	0.863	2.59E-04
mfum_2447	Deoxyribose-phosphate aldolase (EC 4.1.2.4)	2209452	2210097	2.982	-1.578	9.58E-05	-0.797	5.06E-03
mfum_1473	Phosphoglycerate mutase (EC 5.4.2.1)	1361294	1361897	5.366	-1.014	1.05E-04	0.986	4.20E-05
mfum_592	Acetyl-coenzyme A synthetase (EC 6.2.1.1)	562935	564882	6.607	-0.837	5.49E-04	0.124	7.83E-01
mfum_768,mfum_769	NA	734282	735510	4.376	-0.966	9.76E-04	-1.234	2.14E-05
mfum_256	Pyruvate kinase (EC 2.7.1.40)	233808	235239	6.670	-0.769	1.50E-03	-1.309	4.52E-07
mfum_74	6-phosphofructokinase (EC 2.7.1.11)	75481	76573	5.785	-0.742	3.57E-03	-0.219	4.82E-01
mfum_824	Succinate dehydrogenase cytochrome b subunit	778936	779530	4.731	-0.791	4.71E-03	-0.959	1.13E-02
mfum_1967	Fumarate hydratase class II (EC 4.2.1.2)	1802537	1803929	4.878	-0.731	8.23E-03	-0.146	8.33E-01
mfum_1301	Malate dehydrogenase (EC 1.1.1.37)	1219056	1219980	4.846	-0.649	2.03E-02	0.399	1.51E-01
mfum_1729	Succinyl-CoA ligase [ADP-forming] alpha chain (EC 6.2.1.5)	1571852	1572743	6.378	0.506	4.06E-02	-1.372	6.00E-09
mfum_1781	Pyruvate dehydrogenase E1 component alpha subunit (EC 1.2.4.1)	1623349	1624423	7.192	-0.474	5.41E-02	0.231	6.40E-01
mfum_744	Enolase (EC 4.2.1.11)	704076	705351	7.927	0.451	6.21E-02	-1.059	1.25E-05
mfum_731	Acetate kinase (EC 2.7.2.1)	694165	695359	5.044	-0.455	1.04E-01	0.029	1.00E+00
mfum_1852	Isocitrate dehydrogenase [NADP] (EC 1.1.1.42)	1680277	1681723	6.124	-0.371	1.60E-01	0.358	2.79E-01
mfum_825,mfum_826	NA	779545	782259	6.834	-0.353	1.72E-01	0.310	7.62E-01
mfum_1900	Phosphoenolpyruvate carboxykinase [ATP] (EC 4.1.1.49)	1737969	1739502	5.564	-0.312	2.72E-01	-0.486	5.66E-02
mfum_1551	Dihydrolipoamide dehydrogenase (EC 1.8.1.4)	1425438	1426830	4.736	-0.243	4.51E-01	-0.915	1.09E-02
mfum_1782	Pyruvate dehydrogenase E1 component beta subunit (EC 1.2.4.1)	1624470	1625448	6.746	-0.182	5.47E-01	-0.622	1.21E-02
mfum_1730	Succinyl-CoA ligase [ADP-forming] beta chain (EC 6.2.1.5)	1572755	1573940	5.700	-0.091	8.31E-01	-1.899	5.50E-02

Summary statistics for PEGs that are assigned to this pathway are extracted from RAST annotations. RNA-Seq data is previously described [3].

∗ All summary statistics are **log-transformed**. Please note that **CPM**, **FC**, and **FDR** stand for "count-per-million", "fold-change", and "false-discovery-rate".

Supplementary Table 5 – Summary statistics of gene expression for one-carbon metabolism pathway in *M. fumariolicum* SolV under maximum growth (μmax), nitrogen fixation (N_2fix) or oxygen limited (O_2lim) conditions.

		Genomic Position		μmax	N2 fixation		O2 limitation	
**PEGs**	**Function**	**Start**	**End**	**CPM***	**FC***	**FDR***	**FC***	**FDR***
mfum_1141	Particulate methane monooxygenase C-subunit (EC 1.14.13.25)	1066397	1067225	3.242	7.518	2.44E-149	7.597	3.52E-151
mfum_1143	Particulate methane monooxygenase B-subunit (EC 1.14.13.25)	1068069	1069380	4.789	6.339	1.13E-124	6.318	1.04E-123
mfum_1142	Particulate methane monooxygenase A-subunit (EC 1.14.13.25)	1067257	1068058	2.015	6.668	1.17E-111	7.543	8.44E-136
mfum_1150	Particulate methane monooxygenase C-subunit (EC 1.14.13.25)	1075461	1076292	10.306	-5.320	2.74E-96	-6.052	2.96E-113
mfum_1144	Particulate methane monooxygenase C-subunit (EC 1.14.13.25)	1069917	1070748	11.605	-5.107	4.33E-95	-5.724	9.54E-110
mfum_1146	Particulate methane monooxygenase B-subunit (EC 1.14.13.25)	1071623	1072913	10.812	-4.756	2.39E-83	-4.608	5.49E-79
mfum_1145	Particulate methane monooxygenase A-subunit (EC 1.14.13.25)	1070822	1071560	9.953	-4.673	2.08E-79	-4.711	1.23E-79
mfum_1744	NAD-dependent glyceraldehyde-3-phosphate dehydrogenase (EC 1.2.1.12)	1584900	1585944	8.260	-2.301	1.76E-23	-1.716	6.17E-14
mfum_1470	probable RuBisCo-expression protein CbbX	1357219	1358170	9.677	-1.427	2.14E-10	-0.349	1.58E-01
mfum_1796	Methanol dehydrogenase large subunit protein (EC 1.1.99.8)	1636190	1638026	10.342	-1.362	1.23E-09	-0.771	5.25E-03
mfum_1507	NAD-dependent formate dehydrogenase gamma subunit	1391027	1391486	4.556	-1.261	1.15E-05	-3.418	9.09E-05
mfum_1949	Methylenetetrahydrofolate dehydrogenase (NADP+) (EC 1.5.1.5) / Methenyltetrahydrofolate cyclohydrolase (EC 3.5.4.9)	1783786	1784677	4.878	-1.199	1.31E-05	-1.614	5.88E-08
mfum_2186	Serine hydroxymethyltransferase (EC 2.1.2.1)	1966863	1969029	6.872	-0.946	7.30E-05	-0.344	2.05E-01
mfum_1340	Particulate methane monooxygenase C-subunit (EC 1.14.13.25)	1256186	1257050	4.721	-0.915	1.06E-03	-0.656	1.66E-02
mfum_1468	Ribulose bisphosphate carboxylase large chain (EC 4.1.1.39)	1355259	1356723	10.884	-0.734	1.38E-03	-0.369	3.74E-01
mfum_706	Alcohol dehydrogenase (EC 1.1.1.1)	670535	671549	4.986	0.626	1.61E-02	0.152	6.34E-01
mfum_1342	Particulate methane monooxygenase B-subunit (EC 1.14.13.25)	1258072	1259188	1.510	-1.368	2.73E-02	-1.046	1.32E-04
mfum_912	Phosphoribulokinase (EC 2.7.1.19)	865484	866360	6.529	-0.505	4.43E-02	-0.647	8.22E-03
mfum_1116	5-formyltetrahydrofolate cyclo-ligase (EC 6.3.3.2)	1036978	1037572	6.091	-0.452	8.13E-02	-0.077	9.59E-01
mfum_1383	NAD-dependent formate dehydrogenase (EC 1.2.1.2)	1295968	1297165	4.944	-0.445	1.16E-01	-0.802	2.46E-01
mfum_1508,mfum_1509	NA	1391488	1396007	8.940	-0.341	1.72E-01	-0.595	4.90E-02
mfum_810	Formate--tetrahydrofolate ligase (EC 6.3.4.3)	765177	766866	5.916	-0.185	5.55E-01	-0.616	1.45E-02
mfum_1510	NAD-dependent formate dehydrogenase delta subunit	1396069	1396297	4.297	-0.152	7.14E-01	-0.642	6.97E-01
mfum_2215	Phosphoserine aminotransferase (EC 2.6.1.52)	1995104	1996193	8.424	-0.096	7.80E-01	-0.590	5.73E-01
mfum_1341	Particulate methane monooxygenase A-subunit (EC 1.14.13.25)	1257151	1257901	2.389	-0.149	8.90E-01	-0.408	1.00E+00
mfum_1745	Phosphoglycerate kinase (EC 2.7.2.3)	1586020	1587235	7.043	-0.029	1.00E+00	-0.476	6.02E-01

Summary statistics for PEGs that are assigned to this pathway are extracted from RAST annotations. RNA-Seq data is previously described [3].

∗ All summary statistics are **log-transformed**. Please note that **CPM**, **FC**, and **FDR** stand for “count-per-million”, “fold-change”, and “false-discovery-rate”.

Supplementary Table 6 – Summary statistics of gene expression for carbon energy storage pathway in *M. fumariolicum* SolV under maximum growth (μmax), nitrogen fixation (N_2fix) or oxygen limited (O_2lim) conditions.

		Genomic Position		μmax	N2 fixation		O2 limitation	
**PEGs**	**Function**	**Start**	**End**	**CPM***	**FC***	**FDR***	**FC***	**FDR***
mfum_601	Glucose-1-phosphate adenylyltransferase (EC 2.7.7.27)	573176	574484	5.659	-1.385	7.01E-08	-3.387	2.86E-26
mfum_1931	Adenylate kinase (EC 2.7.4.3)	1770361	1770934	4.103	-1.372	8.53E-06	-0.525	5.96E-02
mfum_1365	Glycogen debranching enzyme (EC 3.2.1.-)	1282051	1284193	6.452	-0.959	7.67E-05	-1.000	4.13E-03
mfum_1753,mfum_1754	NA	1595484	1598872	5.736	-0.987	9.71E-05	-2.038	7.52E-07
mfum_829	Phosphate transport system permease protein PstA (TC 3.A.1.7.1)	784281	785121	3.156	-1.255	7.99E-04	-0.761	1.22E-03
mfum_689	Glycogen phosphorylase (EC 2.4.1.1)	652847	655367	7.520	-0.720	2.39E-03	-0.533	5.54E-02
mfum_828	Phosphate transport ATP-binding protein PstB (TC 3.A.1.7.1)	783443	784265	3.451	-1.051	2.53E-03	-0.487	8.99E-02
mfum_830	Phosphate transport system permease protein PstC (TC 3.A.1.7.1)	785123	786149	2.965	-1.137	4.04E-03	-1.637	2.27E-05
mfum_1969	Glycogen phosphorylase (EC 2.4.1.1)	1804825	1806544	4.965	-0.658	1.72E-02	-1.373	5.57E-02
mfum_196	Exopolyphosphatase (EC 3.6.1.11)	179214	180228	6.201	-0.573	2.33E-02	-0.431	1.57E-01
mfum_205	Glycogen synthase, ADP-glucose transglucosylase (EC 2.4.1.21)	189564	191229	5.832	-0.576	2.49E-02	-1.568	1.75E-10
mfum_696	Exopolyphosphatase (EC 3.6.1.11)	660709	662191	4.270	-0.654	2.95E-02	-0.350	2.34E-01
mfum_2070	Glycogen phosphorylase (EC 2.4.1.1)	1880357	1882817	6.994	-0.451	6.97E-02	-1.571	6.59E-07
mfum_1759	Malto-oligosyltrehalose trehalohydrolase (EC 3.2.1.141)	1604384	1606103	4.995	-0.502	7.05E-02	-0.314	3.70E-01
mfum_1219	Polyphosphate kinase (EC 2.7.4.1)	1137650	1139804	6.302	-0.433	9.29E-02	-0.835	4.51E-01
mfum_1898,mfum_1899	NA	1734585	1737805	6.327	-0.223	4.44E-01	-1.349	4.27E-01
mfum_831	Phosphate ABC transporter, periplasmic phosphate-binding protein PstS (TC 3.A.1.7.1)	786169	787303	3.913	-0.215	5.84E-01	-0.221	5.65E-01

Summary statistics for PEGs that are assigned to this pathway are extracted from RAST annotations. RNA-Seq data is previously described [3].

* All summary statistics are **log-transformed**. Please note that **CPM**, **FC**, and **FDR** stand for "count-per-million", "fold-change", and "false-discovery-rate".

Supplementary Table 7 – Summary statistics of gene expression for carbon fixation pathway in *M. fumariolicum* SolV under maximum growth (μmax), nitrogen fixation (N_2fix) or oxygen limited (O_2lim) conditions.

		Genomic Position		μmax	N2 fixation		O2 limitation	
PEGs	Function	Start	End	CPM*	FC*	FDR*	FC*	FDR*
mfum_2211	Ribulose-phosphate 3-epimerase (EC 5.1.3.1)	1992429	1993119	5.765	-1.816	1.69E-12	-0.930	2.47E-04
mfum_1470	probable RuBisCo-expression protein CbbX	1357219	1358170	9.677	-1.427	2.14E-10	-0.349	1.58E-01
mfum_697	Glucose-6-phosphate 1-dehydrogenase (EC 1.1.1.49)	662199	663711	6.847	-1.378	6.87E-09	-0.752	1.15E-02
mfum_1733	hypothetical protein	1577143	1577338	2.804	-2.339	3.45E-07	-0.959	7.89E-04
mfum_1469	Ribulose bisphosphate carboxylase small chain (EC 4.1.1.39)	1356750	1357167	8.992	-1.153	3.88E-07	-1.648	5.20E-01
mfum_625	Ribose 5-phosphate isomerase B (EC 5.3.1.6)	595397	595841	5.232	-1.300	1.03E-06	0.024	1.00E+00
mfum_1637,mfum_1638,mfum_1639,mfum_1640	NA	1498549	1500537	6.504	-1.068	9.82E-06	-2.138	5.69E-21
mfum_626	6-phosphogluconate dehydrogenase, decarboxylating (EC 1.1.1.44)	595879	597295	6.672	-0.986	3.86E-05	-0.905	1.30E-03
mfum_1155	6-phosphogluconolactonase (EC 3.1.1.31), eukaryotic type	1078843	1079512	3.726	-1.162	4.47E-04	0.789	3.49E-02
mfum_1468	Ribulose bisphosphate carboxylase large chain (EC 4.1.1.39)	1355259	1356723	10.884	-0.734	1.38E-03	-0.369	3.74E-01
mfum_1767	Glucose-6-phosphate isomerase (EC 5.3.1.9)	1613654	1615298	5.392	-0.709	6.86E-03	-1.500	9.70E-02
mfum_1885	Xylulose-5-phosphate phosphoketolase (EC 4.1.2.9); Fructose-6-phosphate phosphoketolase (EC 4.1.2.22)	1717483	1719853	6.501	-0.630	1.08E-02	-0.941	3.23E-01
mfum_1472	Transketolase (EC 2.2.1.1)	1359214	1361242	8.985	-0.586	1.28E-02	-2.829	5.21E-04
mfum_1746	Triosephosphate isomerase (EC 5.3.1.1)	1587245	1588013	5.654	-0.588	2.45E-02	-1.626	1.92E-03
mfum_912	Phosphoribulokinase (EC 2.7.1.19)	865484	866360	6.529	-0.505	4.43E-02	-0.647	8.22E-03
mfum_718	Fructose-bisphosphate aldolase class II (EC 4.1.2.13)	680881	681856	8.020	-0.479	4.78E-02	-2.323	6.77E-04
mfum_911	Fructose-1,6-bisphosphatase, type I (EC 3.1.3.11)	864412	865462	7.298	-0.384	1.27E-01	0.368	7.02E-01
mfum_1154	Glucose-6-phosphate 1-dehydrogenase (EC 1.1.1.49)	1077249	1078818	6.649	-0.376	1.46E-01	-2.416	3.40E-24
mfum_2098	Carbonic anhydrase (EC 4.2.1.1)	1902777	1903362	7.814	-0.233	3.93E-01	-0.221	9.30E-01
mfum_1670	transcriptional regulator, LysR family	1522347	1523271	2.284	-0.386	4.97E-01	-1.027	3.67E-05
mfum_2053	Ribose-phosphate pyrophosphokinase (EC 2.7.6.1)	1868494	1869463	6.952	-0.169	5.83E-01	-0.551	3.07E-02
mfum_2433	Ribulose-phosphate 3-epimerase (EC 5.1.3.1)	2196509	2197187	4.156	-0.174	6.60E-01	0.000	1.00E+00
mfum_1313	Fructose-1,6-bisphosphatase, GlpX type (EC 3.1.3.11)	1229380	1230484	6.411	0.100	7.81E-01	-0.545	1.42E-01
mfum_1745	Phosphoglycerate kinase (EC 2.7.2.3)	1586020	1587235	7.043	-0.029	1.00E+00	-0.476	6.02E-01

Summary statistics for PEGs that are assigned to this pathway are extracted from RAST annotations. RNA-Seq data is previously described [3].

∗ All summary statistics are **log-transformed**. Please note that **CPM**, **FC**, and **FDR** stand for "count-per-million", "fold-change", and "false-discovery-rate".

Supplementary Table 8 – Summary statistics of gene expression for nitrogen metabolism pathway in *M. fumariolicum* SolV under maximum growth (μmax), nitrogen fixation (N_2fix) or oxygen limited (O_2lim) conditions.

		Genomic Position		μmax	N2 fixation		O2 limitation	
**PEGs**	**Function**	**Start**	**End**	**CPM***	**FC***	**FDR***	**FC***	**FDR***
mfum_1695	Ammonium transporter	1547869	1549402	5.672	1.906	2.59E-16	-0.486	6.70E-02
mfum_398	Hydroxylamine oxidoreductase precursor (EC 1.7.3.4)	364936	367084	6.455	-1.645	1.32E-11	0.136	6.67E-01
mfum_314,mfum_315	NA	284396	286128	5.353	-1.388	1.37E-07	-0.042	9.66E-01
mfum_1671	Nitrate ABC transporter, nitrate-binding protein	1523369	1524452	2.048	1.573	4.56E-06	-1.189	1.84E-04
mfum_781	transcriptional regulator, NifA subfamily, Fis Family	744180	745632	6.737	-0.865	3.21E-04	-1.001	3.05E-02
mfum_1672,mfum_1673,mfum_1674	NA	1524783	1529475	2.726	1.123	4.24E-04	-1.210	4.21E-03
mfum_316	Nitric-oxide reductase subunit C (EC 1.7.99.7)	286137	286812	3.726	-1.140	5.69E-04	-0.026	1.00E+00
mfum_1547	Glutamine synthetase type I (EC 6.3.1.2)	1423025	1424459	7.952	-0.736	1.70E-03	-0.088	8.16E-01
mfum_52	Response regulator of zinc sigma-54-dependent two-component system	56365	57751	5.203	-0.740	5.48E-03	-0.258	5.49E-01
mfum_1099	[Protein-PII] uridylyltransferase (EC 2.7.7.59)	1017048	1019763	5.690	-0.636	1.42E-02	0.103	8.79E-01
mfum_2364	dienelactone hydrolase family protein	2135493	2136450	4.755	-0.647	2.19E-02	-0.005	1.00E+00
mfum_985	Carbamoyl-phosphate synthase small chain (EC 6.3.5.5)	931643	932855	6.110	-0.564	2.63E-02	-0.953	3.79E-01
mfum_1010,mfum_1011	NA	955688	957616	6.978	-0.540	2.75E-02	-1.254	1.25E-03
mfum_2641	Membrane protein, distant similarity to thiosulphate:quinone oxidoreductase DoxD	2385131	2385602	3.256	-0.716	4.87E-02	-0.470	2.16E-01
mfum_405	Argininosuccinate lyase (EC 4.3.2.1)	372872	374267	5.270	-0.527	5.03E-02	0.004	1.00E+00
mfum_1686	Nitrogen regulatory protein P-II	1538385	1538766	2.913	-0.728	7.06E-02	-1.665	1.52E-05
mfum_1548	Nitrogen regulatory protein P-II	1424652	1424997	5.789	0.349	1.95E-01	-0.593	1.83E-02
mfum_1675	nitrite reductase [NAD(P)H], small subunit(EC:1.7.1.4)	1529497	1529857	0.463	-0.739	4.78E-01	-0.708	3.08E-03
mfum_929	Copper-containing nitrite reductase (EC 1.7.2.1)	883173	884538	3.578	-0.258	5.08E-01	0.162	5.93E-01
mfum_1676,mfum_1677	NA	1529865	1533587	3.171	-0.239	5.74E-01	-1.422	6.76E-08
mfum_474	Glutamate synthase [NADPH] large chain (EC 1.4.1.13)	437507	442067	9.448	-0.131	6.66E-01	-1.310	1.89E-01
mfum_1755	sigma-54 dependent transcriptional regulator/response regulator	1598883	1600278	4.163	-0.149	7.26E-01	-0.530	2.85E-01
mfum_1836	Aminotransferase HpnO, required for aminobacteriohopanetriol	1669794	1671192	6.840	-0.060	9.10E-01	-1.517	1.04E-06
mfum_210	Acetylornithine aminotransferase (EC 2.6.1.11)	195322	196546	5.619	-0.050	9.58E-01	0.070	8.81E-01
mfum_209	Ornithine carbamoyltransferase (EC 2.1.3.3)	194324	195242	4.751	-0.018	1.00E+00	-2.132	1.21E-04
mfum_845	Alanine dehydrogenase (EC 1.4.1.1)	794186	795359	5.075	-0.010	1.00E+00	-0.537	7.98E-02

Summary statistics for PEGs that are assigned to this pathway are extracted from RAST annotations. RNA-Seq data is previously described [3].

∗ All summary statistics are **log-transformed**. Please note that **CPM**, **FC**, and **FDR** stand for "count-per-million", "fold-change", and "false-discovery-rate".

Supplementary Table 9 – Summary statistics of gene expression for nitrogen fixation pathway in *M. fumariolicum* SolV under maximum growth (μmax), nitrogen fixation (N_2fix) or oxygen limited (O_2lim) conditions.

		Genomic Position		μmax	N2 fixation		O2 limitation	
**PEGs**	**Function**	**Start**	**End**	**CPM***	**FC***	**FDR***	**FC***	**FDR***
mfum_2509	Nitrogenase (molybdenum-iron) reductase and maturation protein NifH	2258348	2259242	2.878	8.347	4.51E-170	-0.353	4.53E-01
mfum_2508	Nitrogenase (molybdenum-iron) alpha chain (EC 1.18.6.1)	2256760	2258236	3.813	7.223	2.56E-145	-1.076	1.17E-03
mfum_2496	Nitrogenase FeMo-cofactor synthesis FeS core scaffold and assembly protein NifB	2245737	2247306	1.726	7.563	3.28E-133	-0.780	1.95E-01
mfum_2507	Nitrogenase (molybdenum-iron) beta chain (EC 1.18.6.1)	2255136	2256717	3.048	6.143	5.28E-108	-0.548	1.72E-01
mfum_2506	Nitrogenase FeMo-cofactor scaffold and assembly protein NifE	2253714	2255094	3.905	5.822	1.61E-105	-0.890	5.10E-03
mfum_2505	Nitrogenase FeMo-cofactor scaffold and assembly protein NifN	2252355	2253699	3.389	5.616	3.88E-96	-0.765	3.04E-02
mfum_2495	4Fe-4S ferredoxin, nitrogenase-associated	2245484	2245709	2.284	5.971	1.34E-95	-0.943	4.09E-02
mfum_2479	Electron transfer flavoprotein, beta subunit	2233843	2234683	3.475	5.376	3.59E-90	-0.324	3.86E-01
mfum_2476,mfum_2477,mfum_2478	NA	2231060	2233761	4.905	5.031	4.07E-88	-0.534	5.88E-02
mfum_2494	probable iron binding protein from the HesB_IscA_SufA family in Nif operon	2245140	2245482	0.948	5.735	7.63E-71	-1.068	2.06E-01
mfum_2504	Nitrogenase FeMo-cofactor carrier protein NifX	2251953	2252352	2.487	4.736	2.18E-64	-2.076	2.56E-05
mfum_2480	Nitrogenase stabilizing/protective protein NifW	2234695	2235103	2.200	4.751	3.33E-62	-1.587	1.87E-03
mfum_2503	NifX-associated protein	2251478	2251949	2.913	4.326	6.31E-58	-0.339	4.64E-01
mfum_2490,mfum_2491,mfum_2492,mfum_2493	NA	2242330	2245064	5.171	3.690	3.17E-53	-1.259	3.19E-06
mfum_2484	NifT protein	2237497	2237707	2.110	3.555	5.69E-34	-1.089	2.95E-02
mfum_2487	Nitrogenase (molybdenum-iron)-specific transcriptional regulator NifA	2238817	2240407	4.700	2.918	6.45E-34	-0.634	2.62E-02
mfum_1385,mfum_1386	NA	1297489	1300400	5.398	2.126	1.09E-19	3.614	1.70E-51
mfum_1387,mfum_1388	NA	1300411	1301807	4.110	2.171	3.50E-18	2.379	2.05E-21
mfum_361	Iron-sulfur cluster assembly protein SufD	334497	335790	6.577	1.570	6.06E-12	-1.716	2.18E-12
mfum_2489	Nitrogenase FeMo-cofactor synthesis molybdenum delivery protein NifQ	2241694	2242288	3.228	1.835	3.73E-11	-1.531	6.30E-05
mfum_728	Putative iron-sulfur cluster assembly scaffold protein for SUF system, SufE2	690804	691227	1.948	-3.330	8.59E-07	-2.178	3.63E-15
mfum_2511,mfum_2512	NA	2259497	2260740	4.110	1.182	7.09E-06	-0.923	1.31E-04
mfum_2359	probable iron binding protein from the HesB_IscA_SufA family	2131915	2132257	2.487	-1.932	5.40E-05	-0.516	5.82E-02
mfum_362	Iron-sulfur cluster assembly protein SufB	335836	337261	7.583	0.907	7.99E-05	-0.565	1.85E-02
mfum_1628	Fe-S metabolism associated SufE	1490830	1491298	4.344	-1.049	3.65E-04	-1.233	9.30E-02
mfum_2116	Glucans biosynthesis protein G precursor	1912853	1914383	4.304	-0.894	2.53E-03	-0.812	5.00E-04
mfum_729	Cysteine desulfurase (EC 2.8.1.7), SufS subfamily	691242	692472	4.982	-0.821	2.62E-03	-0.655	6.10E-02
mfum_1997,mfum_1998	NA	1825557	1828268	6.282	-0.622	1.30E-02	-0.799	7.40E-03
mfum_1818	Cysteine desulfurase (EC 2.8.1.7)	1654069	1655224	6.478	-0.593	1.71E-02	-0.211	8.33E-01
mfum_356	Cysteine desulfurase (EC 2.8.1.7)	329595	330732	4.918	0.592	2.45E-02	-1.503	9.27E-10
mfum_2000	[NiFe] hydrogenase nickel incorporation-associated protein HypB	1829063	1829864	5.896	-0.537	3.70E-02	1.127	1.70E-02
mfum_1996	[NiFe] hydrogenase metallocenter assembly protein HypD	1824404	1825517	7.285	-0.487	4.67E-02	-1.420	5.67E-02
mfum_1994,mfum_1995	NA	1822140	1824356	6.698	0.383	1.30E-01	-2.059	5.86E-04

mfum_2111	[NiFe] hydrogenase nickel incorporation protein HybF	1907980	1908322	2.389	-0.562	2.48E-01	-0.334	2.34E-01
mfum_454	Pyruvate-flavodoxin oxidoreductase (EC 1.2.7.-)	417964	421513	5.926	-0.140	6.69E-01	-0.185	5.69E-01
mfum_1543,mfum_1544	NA	1418446	1420413	4.731	-0.148	6.82E-01	-0.654	1.66E-02

Summary statistics for PEGs that are assigned to this pathway are extracted from RAST annotations. RNA-Seq data is previously described [3].

∗ All summary statistics are **log-transformed**. Please note that **CPM**, **FC**, and **FDR** stand for “count-per-million”, “fold-change”, and “false-discovery-rate”.

Supplementary Table 10 – Summary statistics of gene expression for methane oxidation pathway in *M. fumariolicum* SolV under maximum growth (μmax), nitrogen fixation (N_2fix) or oxygen limited (O_2lim) conditions.

PEGs	Function	Genomic Position Start	End	μmax CPM*	N2 fixation FC*	FDR*	O2 limitation FC*	FDR*
mfum_1141	Particulate methane monooxygenase C-subunit (EC 1.14.13.25)	1066397	1067225	3.242	7.518	2.44E-149	7.597	3.52E-151
mfum_1143	Particulate methane monooxygenase B-subunit (EC 1.14.13.25)	1068069	1069380	4.789	6.339	1.13E-124	6.318	1.04E-123
mfum_1142	Particulate methane monooxygenase A-subunit (EC 1.14.13.25)	1067257	1068058	2.015	6.668	1.17E-111	7.543	8.44E-136
mfum_1150	Particulate methane monooxygenase C-subunit (EC 1.14.13.25)	1075461	1076292	10.306	-5.320	2.74E-96	-6.052	2.96E-113
mfum_1144	Particulate methane monooxygenase C-subunit (EC 1.14.13.25)	1069917	1070748	11.605	-5.107	4.33E-95	-5.724	9.54E-110
mfum_1146	Particulate methane monooxygenase B-subunit (EC 1.14.13.25)	1071623	1072913	10.812	-4.756	2.39E-83	-4.608	5.49E-79
mfum_1145	Particulate methane monooxygenase A-subunit (EC 1.14.13.25)	1070822	1071560	9.953	-4.673	2.08E-79	-4.711	1.23E-79
mfum_1796	Methanol dehydrogenase large subunit protein (EC 1.1.99.8)	1636190	1638026	10.342	-1.362	1.23E-09	-0.771	5.25E-03
mfum_1507	NAD-dependent formate dehydrogenase gamma subunit	1391027	1391486	4.556	-1.261	1.15E-05	-3.418	9.09E-05
mfum_1949	Methylenetetrahydrofolate dehydrogenase (NADP+) (EC 1.5.1.5) / Methenyltetrahydrofolate cyclohydrolase (EC 3.5.4.9)	1783786	1784677	4.878	-1.199	1.31E-05	-1.614	5.88E-08
mfum_273	GTP cyclohydrolase I (EC 3.5.4.16) type 2	253507	254329	6.418	-1.050	1.48E-05	-1.619	2.88E-04
mfum_1502	Coenzyme PQQ synthesis protein E	1386330	1387353	5.965	0.895	1.83E-04	-4.383	1.85E-09
mfum_2298,mfum_2299	NA	2073229	2074479	4.544	-0.983	6.14E-04	-2.962	1.63E-05
mfum_1505	Coenzyme PQQ synthesis protein B	1388525	1389422	5.622	-0.859	8.04E-04	-0.781	2.42E-02
mfum_1340	Particulate methane monooxygenase C-subunit (EC 1.14.13.25)	1256186	1257050	4.721	-0.915	1.06E-03	-0.656	1.66E-02
mfum_706	Alcohol dehydrogenase (EC 1.1.1.1)	670535	671549	4.986	0.626	1.61E-02	0.152	6.34E-01
mfum_1342	Particulate methane monooxygenase B-subunit (EC 1.14.13.25)	1258072	1259188	1.510	-1.368	2.73E-02	-1.046	1.32E-04
mfum_2706,mfum_2707,mfum_2708	NA	2446024	2447755	3.931	-0.610	5.36E-02	0.254	3.86E-01
mfum_1383	NAD-dependent formate dehydrogenase (EC 1.2.1.2)	1295968	1297165	4.944	-0.445	1.16E-01	-0.802	2.46E-01
mfum_2539,mfum_2540	NA	2282337	2283941	5.235	-0.435	1.19E-01	-0.239	1.00E+00
mfum_2705	hypothetical protein	2445896	2446037	0.015	-1.808	1.21E-01	-0.240	3.93E-01
mfum_973	Alcohol dehydrogenase (EC 1.1.1.1)	919658	920687	5.337	-0.424	1.23E-01	-4.330	1.20E-01
mfum_2435	hypothetical protein	2199064	2199229	0.948	-1.069	1.63E-01	0.009	1.00E+00
mfum_470	Dihydropteroate synthase (EC 2.5.1.15)	434115	435018	4.163	0.414	1.71E-01	-1.411	1.32E-06
mfum_1508,mfum_1509	NA	1391488	1396007	8.940	-0.341	1.72E-01	-0.595	4.90E-02
mfum_810	Formate--tetrahydrofolate ligase (EC 6.3.4.3)	765177	766866	5.916	-0.185	5.55E-01	-0.616	1.45E-02
mfum_803	Quinone oxidoreductase (EC 1.6.5.5)	756842	757802	6.105	0.120	7.31E-01	0.449	2.24E-01
mfum_2412	NAD(P)HX epimerase / NAD(P)HX dehydratase	2181264	2182827	5.763	0.100	7.99E-01	-0.460	5.72E-02
mfum_355	Serine acetyltransferase (EC 2.3.1.30)	328707	329580	5.095	-0.105	8.01E-01	-0.612	1.41E-02
mfum_1503,mfum_1504	NA	1387467	1388515	5.646	0.077	8.69E-01	-2.446	2.65E-06
mfum_1341	Particulate methane monooxygenase A-subunit (EC 1.14.13.25)	1257151	1257901	2.389	-0.149	8.90E-01	-0.408	1.00E+00
mfum_462	Aldehyde dehydrogenase (EC 1.2.1.3)	428152	429649	3.556	-0.029	1.00E+00	-0.602	1.99E-02

Summary statistics for PEGs that are assigned to this pathway are extracted from RAST annotations. RNA-Seq data is previously described [3].

* All summary statistics are **log-transformed**. Please note that **CPM**, **FC**, and **FDR** stand for "count-per-million", "fold-change", and "false-discovery-rate".

Supplementary Table 11 – Summary statistics of gene expression for glycogen metabolism pathway in *M. fumariolicum* SolV under maximum growth (μmax), nitrogen fixation (N_2fix) or oxygen limited (O_2lim) conditions.

		Genomic Position		μmax	N2 fixation		O2 limitation	
**PEGs**	**Function**	**Start**	**End**	**CPM***	**FC***	**FDR***	**FC***	**FDR***
mfum_601	Glucose-1-phosphate adenylyltransferase (EC 2.7.7.27)	573176	574484	5.659	-1.385	7.01E-08	-3.387	2.86E-26
mfum_1365	Glycogen debranching enzyme (EC 3.2.1.-)	1282051	1284193	6.452	-0.959	7.67E-05	-1.000	4.13E-03
mfum_1753,mfum_1754	NA	1595484	1598872	5.736	-0.987	9.71E-05	-2.038	7.52E-07
mfum_96	Phosphomannomutase (EC 5.4.2.8)	97170	99099	6.398	-0.807	9.81E-04	-0.400	1.65E-01
mfum_689	Glycogen phosphorylase (EC 2.4.1.1)	652847	655367	7.520	-0.720	2.39E-03	-0.533	5.54E-02
mfum_205	Glycogen synthase, ADP-glucose transglucosylase (EC 2.4.1.21)	189564	191229	5.832	-0.576	2.49E-02	-1.568	1.75E-10
mfum_2070	Glycogen phosphorylase (EC 2.4.1.1)	1880357	1882817	6.994	-0.451	6.97E-02	-1.571	6.59E-07
mfum_1898,mfum_1899	NA	1734585	1737805	6.327	-0.223	4.44E-01	-1.349	4.27E-01
mfum_1848	4-alpha-glucanotransferase (amylomaltase) (EC 2.4.1.25)	1677042	1678737	4.561	0.075	9.13E-01	0.128	1.00E+00
mfum_1969	Glycogen phosphorylase (EC 2.4.1.1)	1804825	1806544	4.965	-0.658	1.72E-02	-1.373	5.57E-02

Summary statistics for PEGs that are assigned to this pathway are extracted from RAST annotations. RNA-Seq data is previously described [3].

* All summary statistics are **log-transformed**. Please note that **CPM**, **FC**, and **FDR** stand for “count-per-million”, “fold-change”, and “false-discovery-rate”.

Supplementary Table 12 – Summary statistics of gene expression for Calvin Benson Bassham (CBB) cycle pathway in *M. fumariolicum* SolV under maximum growth (μmax), nitrogen fixation (N_2fix) or oxygen limited (O_2lim) conditions.

		Genomic Position		μmax	N2 fixation		O2 limitation	
**PEGs**	**Function**	**Start**	**End**	**CPM***	**FC***	**FDR***	**FC***	**FDR***
mfum_1744	NAD-dependent glyceraldehyde-3-phosphate dehydrogenase (EC 1.2.1.12)	1584900	1585944	8.260	-2.301	1.76E-23	-1.716	6.17E-14
mfum_2211	Ribulose-phosphate 3-epimerase (EC 5.1.3.1)	1992429	1993119	5.765	-1.816	1.69E-12	-0.930	2.47E-04
mfum_697	Glucose-6-phosphate 1-dehydrogenase (EC 1.1.1.49)	662199	663711	6.847	-1.378	6.87E-09	-0.752	1.15E-02
mfum_1469	Ribulose bisphosphate carboxylase small chain (EC 4.1.1.39)	1356750	1357167	8.992	-1.153	3.88E-07	-1.648	5.20E-01
mfum_625	Ribose 5-phosphate isomerase B (EC 5.3.1.6)	595397	595841	5.232	-1.300	1.03E-06	0.024	1.00E+00
mfum_1471	Uridine kinase (EC 2.7.1.48)	1358202	1359210	7.798	-1.031	8.23E-06	-0.376	4.02E-01
mfum_1637,mfum_1638,mfum_1639,mfum_1640	NA	1498549	1500537	6.504	-1.068	9.82E-06	-2.138	5.69E-21
mfum_626	6-phosphogluconate dehydrogenase, decarboxylating (EC 1.1.1.44)	595879	597295	6.672	-0.986	3.86E-05	-0.905	1.30E-03
mfum_1155	6-phosphogluconolactonase (EC 3.1.1.31), eukaryotic type	1078843	1079512	3.726	-1.162	4.47E-04	0.789	3.49E-02
mfum_93	Fructose-bisphosphate aldolase, archaeal class I (EC 4.1.2.13)	94110	95028	4.751	-0.954	6.14E-04	-0.495	5.56E-02
mfum_1468	Ribulose bisphosphate carboxylase large chain (EC 4.1.1.39)	1355259	1356723	10.884	-0.734	1.38E-03	-0.369	3.74E-01
mfum_1767	Glucose-6-phosphate isomerase (EC 5.3.1.9)	1613654	1615298	5.392	-0.709	6.86E-03	-1.500	9.70E-02
mfum_1885	Xylulose-5-phosphate phosphoketolase (EC 4.1.2.9); Fructose-6-phosphate phosphoketolase [illegible]	1717483	1719853	6.501	-0.630	1.08E-02	-0.941	3.23E-01
mfum_1472	Transketolase (EC 2.2.1.1)	1359214	1361242	8.985	-0.586	1.28E-02	-2.829	5.21E-04
mfum_1746	Triosephosphate isomerase (EC 5.3.1.1)	1587245	1588013	5.654	-0.588	2.45E-02	-1.626	1.92E-03
mfum_912	Phosphoribulokinase (EC 2.7.1.19)	865484	866360	6.529	-0.505	4.43E-02	-0.647	8.22E-03
mfum_718	Fructose-bisphosphate aldolase class II (EC 4.1.2.13)	680881	681856	8.020	-0.479	4.78E-02	-2.323	6.77E-04
mfum_911	Fructose-1,6-bisphosphatase, type I (EC 3.1.3.11)	864412	865462	7.298	-0.384	1.27E-01	0.368	7.02E-01
mfum_1154	Glucose-6-phosphate 1-dehydrogenase (EC 1.1.1.49)	1077249	1078818	6.649	-0.376	1.46E-01	-2.416	3.40E-24
mfum_2433	Ribulose-phosphate 3-epimerase (EC 5.1.3.1)	2196509	2197187	4.156	-0.174	6.60E-01	0.000	1.00E+00
mfum_1313	Fructose-1,6-bisphosphatase, GlpX type (EC 3.1.3.11)	1229380	1230484	6.411	0.100	7.81E-01	-0.545	1.42E-01
mfum_1745	Phosphoglycerate kinase (EC 2.7.2.3)	1586020	1587235	7.043	-0.029	1.00E+00	-0.476	6.02E-01

Summary statistics for PEGs that are assigned to this pathway are extracted from RAST annotations. RNA-Seq data is previously described [3].

* All summary statistics are **log-transformed**. Please note that **CPM**, **FC**, and **FDR** stand for "count-per-million", "fold-change", and "false-discovery-rate".

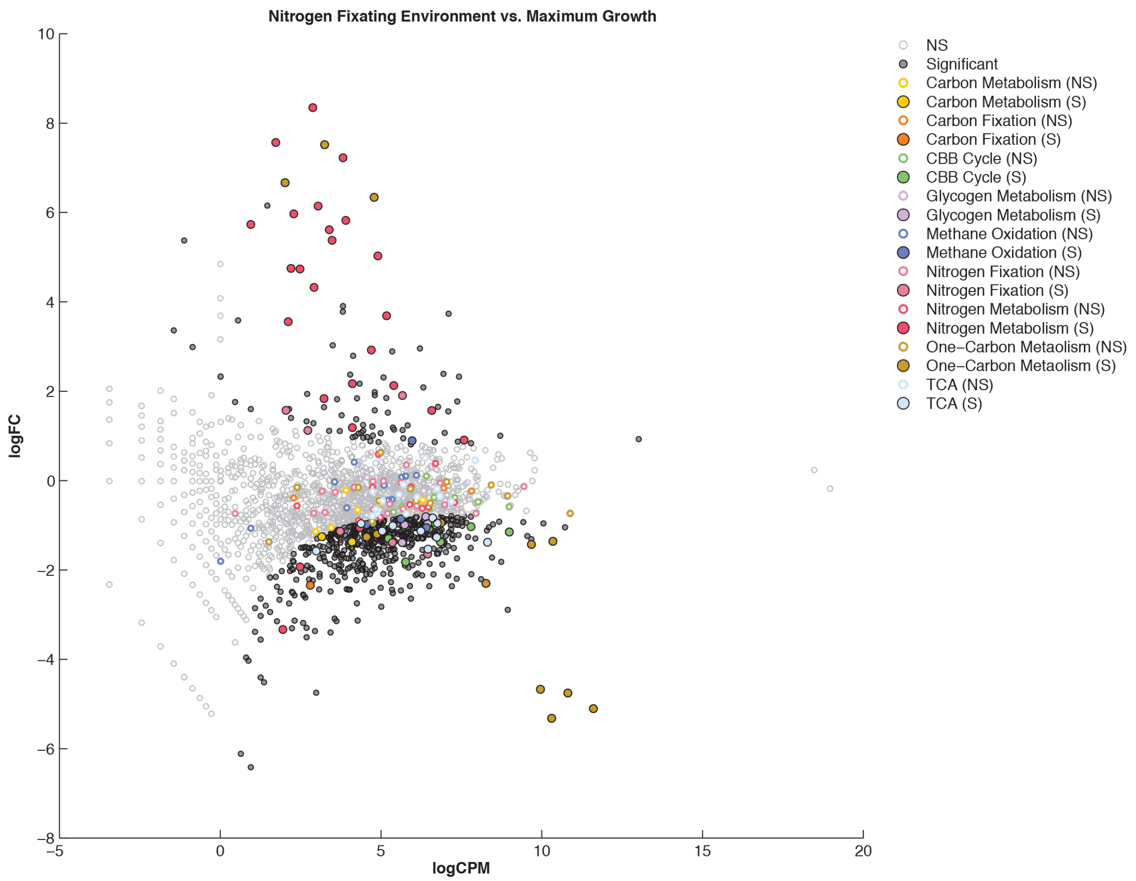

Supplementary Figure 13 – MA plot of deregulated genes in cells under nitrogen fixing environment compared to cells that are at maximum growth. All annotated genes in the genome of *Methylacidiphilum fumariolicum* SolV are depicted as deregulated (filled circles; noted by 'S' in the legends) or not significant (empty circles; noted by 'NS' in the legends). The colouring is based on their associated pathway. CPM and FC stand for count-per-million and fold-change, respectively.

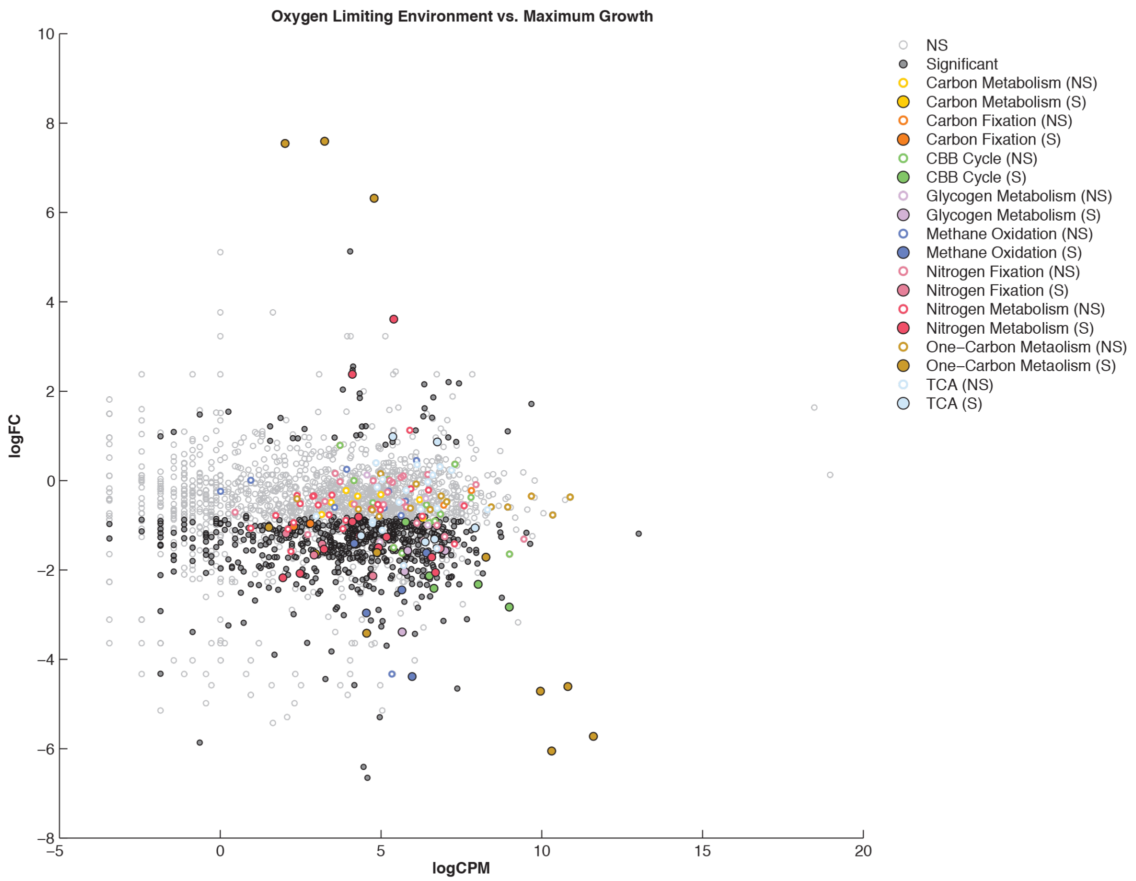

Supplementary Figure 14 – MA plot of deregulated genes in cells under oxygen limitation environment compared to cells that are at maximum growth. All annotated genes in the genome of *Methylacidiphilum fumariolicum* SolV are depicted as deregulated (filled circles; noted by 'S' in the legends) or not significant (empty circles; noted by 'NS' in the legends). The colouring is based on their associated pathway. CPM and FC stand for count-per-million and fold change, respectively.

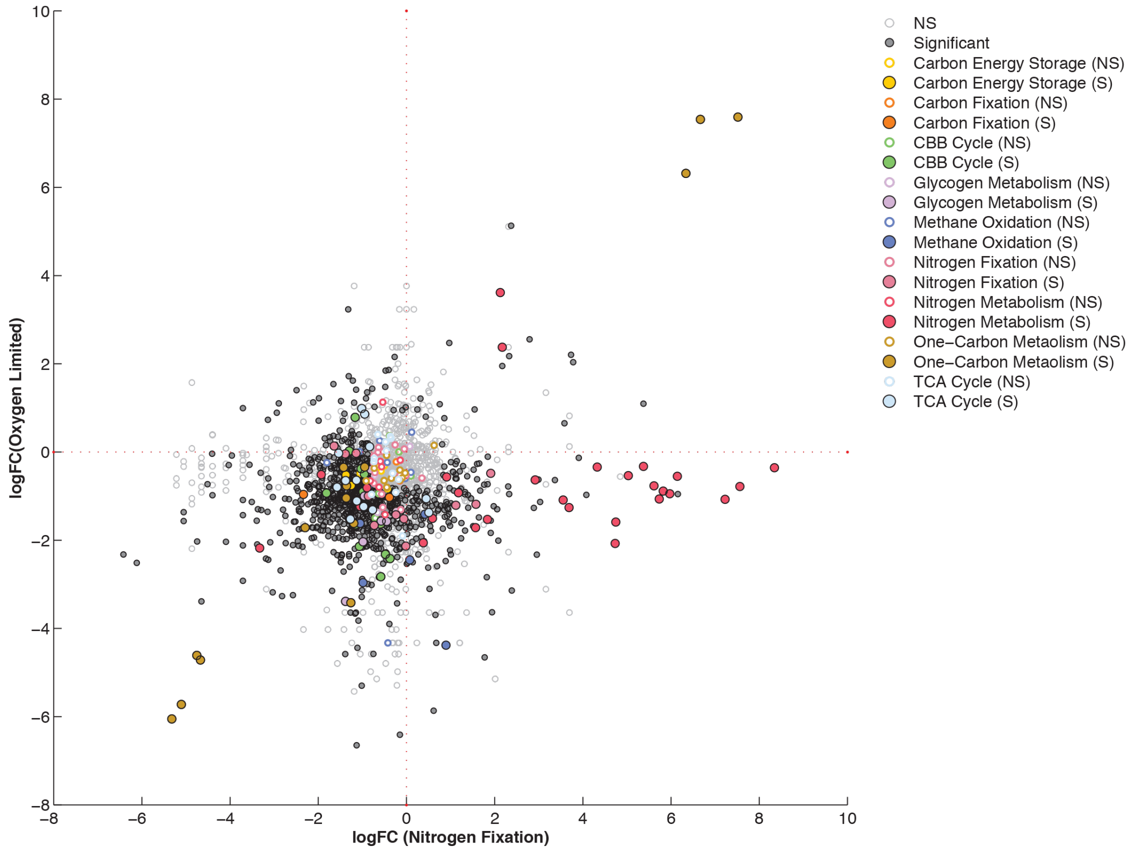

Supplementary Figure 15 – Relative gene expression fold change between cells under nitrogen fixing and oxygen limitation environments as compared to cells that are at maximum growth. All annotated genes in the genome of *Methylacidiphilum fumariolicum* SolV are depicted as deregulated (filled circles; noted by 'S' in the legends) or not significant (empty circles; noted by 'NS' in the legends). The colouring is based on their associated pathway. FC stands for fold change.

Supplementary Table 13 – A list of annotated DNA/RNA methyltransferases in *Methylacidiphilum fumariolicum* SolV genome. All analyses were performed using RAST functional annotation and comparative analysis.

**PEG ID**	**Subsystem**	**Role**	**Identity to *M. infernorum* V4**
mfum.peg.88	DNA repair	Methylated-DNA--protein-cysteine methyltransferase (EC 2.1.1.63)	58.3% (peg.2204)
Mfum.peg.128	Restriction-Modification System	Type I restriction-modification system, restriction subunit R (EC 3.1.21.3)	NA
Mfum.peg.129	Restriction-Modification System	Type I restriction-modification system, specificity subunit S (EC 3.1.21.3)	NA
mfum.peg.130	Restriction-Modification System	Type I restriction-modification system, DNA-methyltransferase subunit M (EC 2.1.1.72)	NA
mfum.peg.145	-	Methyltransferase type 11	75.8% (peg.142)
mfum.peg.471	-	Methyltransferase FkbM family	57.4% (peg.2380)
mfum.peg.753	-	Putative DNA modification methylase	NA
mfum.peg.1363	-	Methyltransferase (EC 2.1.1.-)	54.1% (peg.48)
mfum.peg.1422	-	Methyltransferase (EC 2.1.1.-)	61.7% (peg.1841)
mfum.peg.1659	-	Methyltransferase type 12	64.3% (peg.1162)
mfum.peg.2043	-	Putative methyltransferase	68.0% (peg.707)
mfum.peg.2101	-	Methyltransferase (EC 2.1.1.-)	47.0% (peg.792)
mfum.peg.2555	-	Methyltransferase type 11	52.1% (peg.585)
mfum.peg.2561	-	Methyltransferase FkbM	56.8% (peg.563)
mfum.peg.2564	-	Methyltransferase	52.4% (peg.580)
mfum.peg.2632	-	Methyltransferase type 11	67.7% (peg.1987)
mfum.peg.160	-	RNA methyltransferase, TrmA family	64.5% (peg.134)
mfum.peg.252	RNA methylation	Ribosomal RNA large subunit methyltransferase N (EC 2.1.1.-)	72.5% (peg.85)
mfum.peg.337	RNA methylation	LSU m5C1962 methyltransferase RlmI	78.0% (peg.18)
mfum.peg.481	RNA methylation	tRNA (cytidine(34)-2'-O)-methyltransferase (EC 2.1.1.207)	75.2% (peg.2354)
mfum.peg.489	RNA methylation	tRNA (guanine46-N7-)-methyltransferase (EC 2.1.1.33)	58.8% (peg.2347)
mfum.peg.1451	RNA methylation	tRNA (Guanine37-N1) -methyltransferase (EC 2.1.1.31)	80.3% (peg.1326)
mfum.peg.2445	RNA methylation	Ribosomal RNA small subunit methyltransferase E (EC 2.1.1.-)	60.7% (peg.1903)

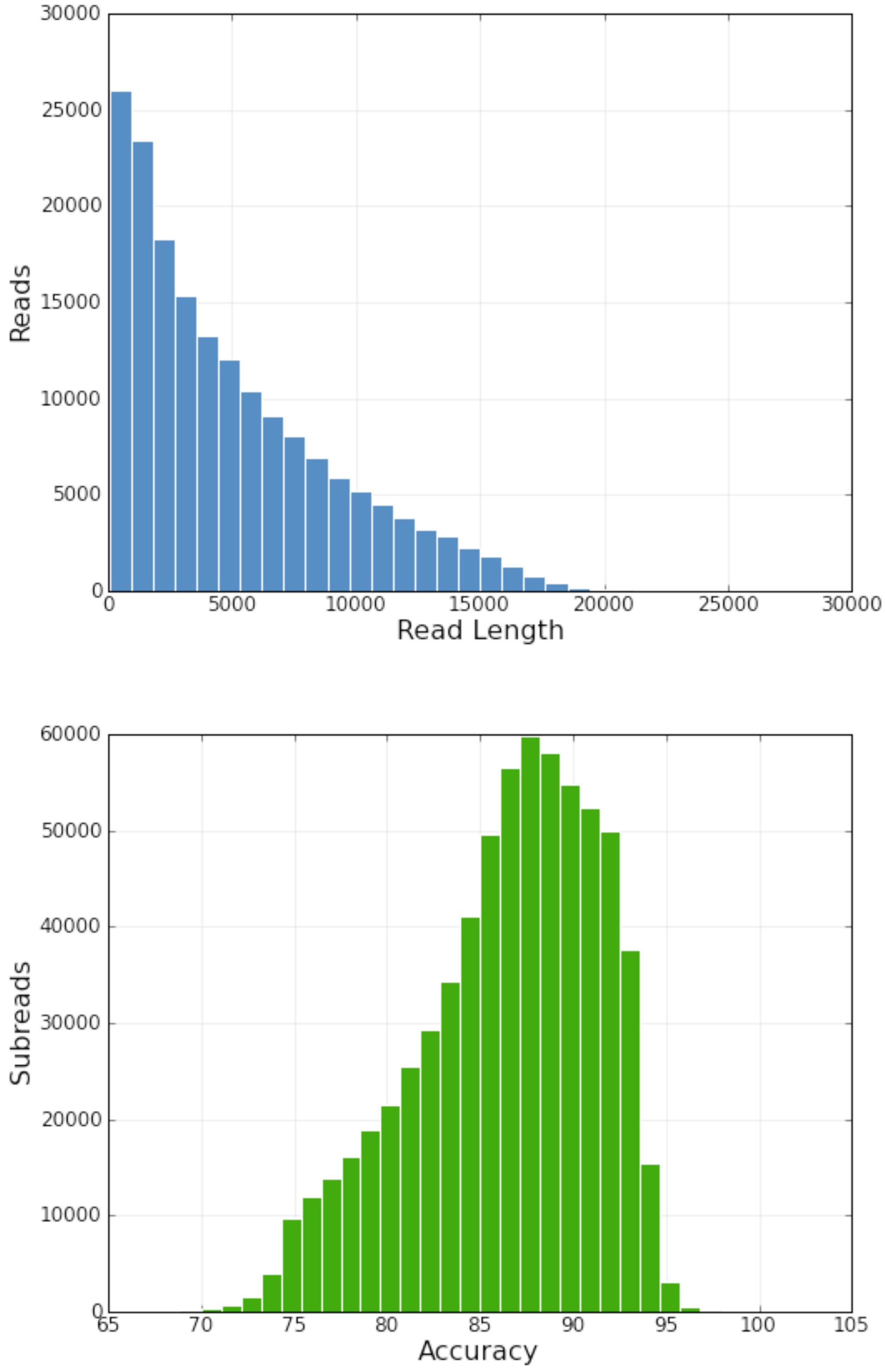

Supplementary Figure 16 – Read length and accuracy histograms of reads that map to the genome of *Methylacidiphilum fumariolicum* SolV. Reads from two TET-treated SMRT sequencing runs are used to identify methylated bases and associated motifs.

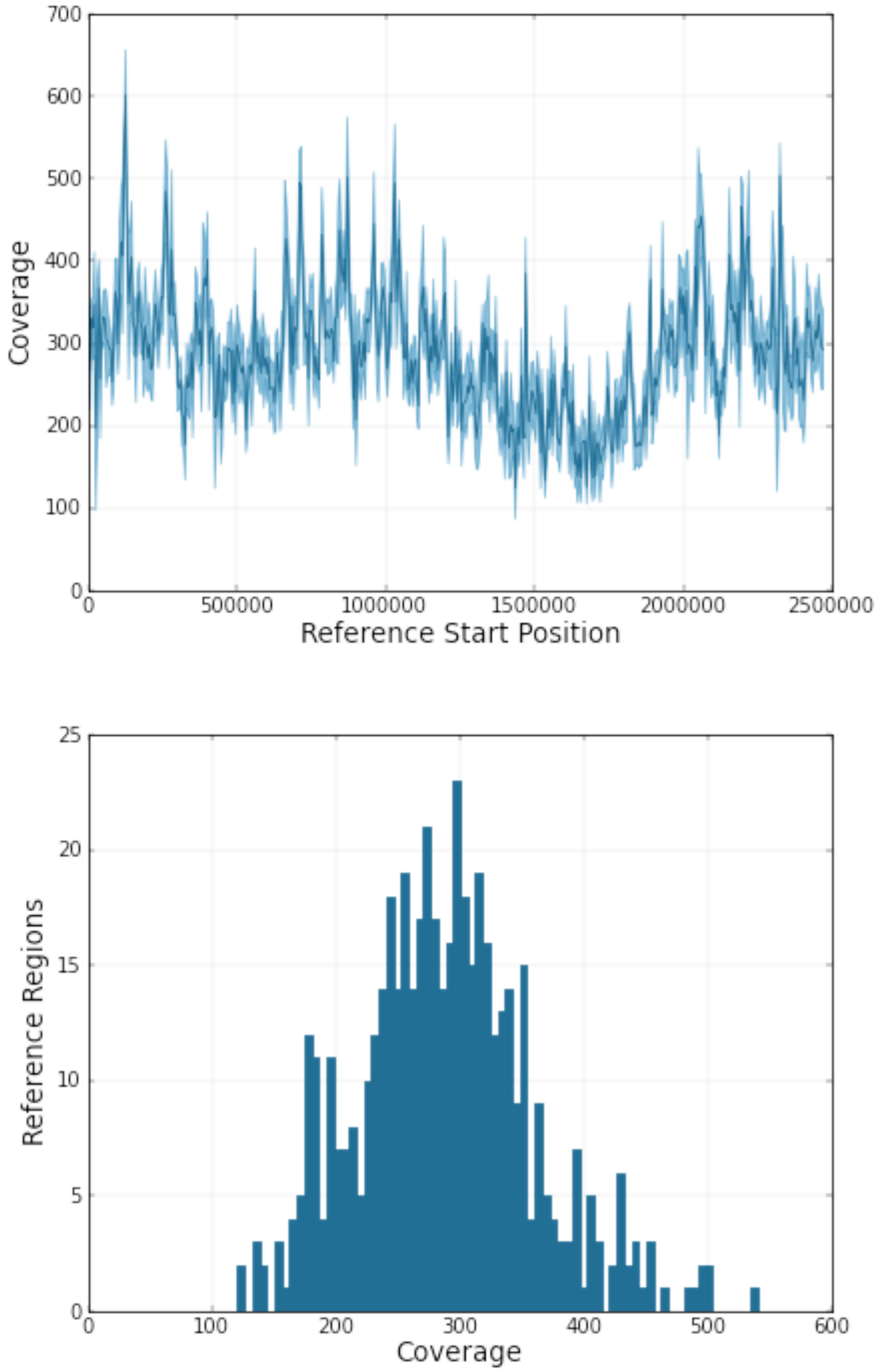

Supplementary Figure 17 – Coverage profile and distribution across the *Methylacidiphilum fumariolicum* SolV genome. Reads from two TET-treated SMRT sequencing runs are used to identify methylated bases and associated motifs.

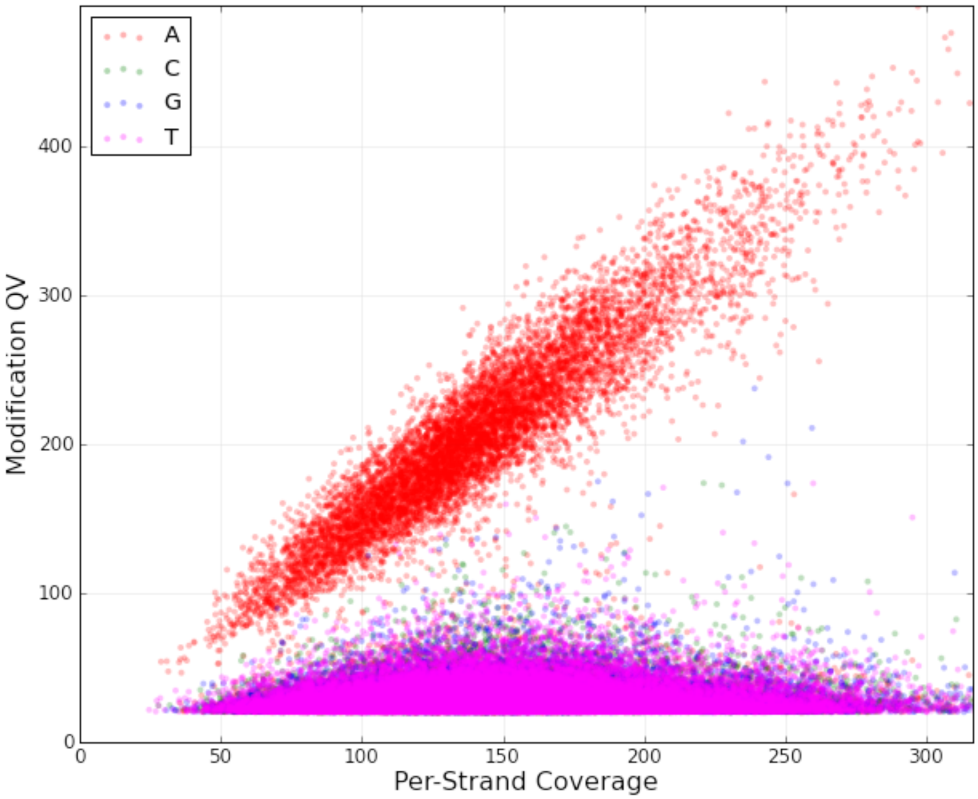

Supplementary Figure 18 – Modification quality values compared to coverage per strand. Average quality values for modification of each base in the *Methylacidiphilum fumariolicum* SolV genome are compared to the strand-specific coverage at each genomic position.

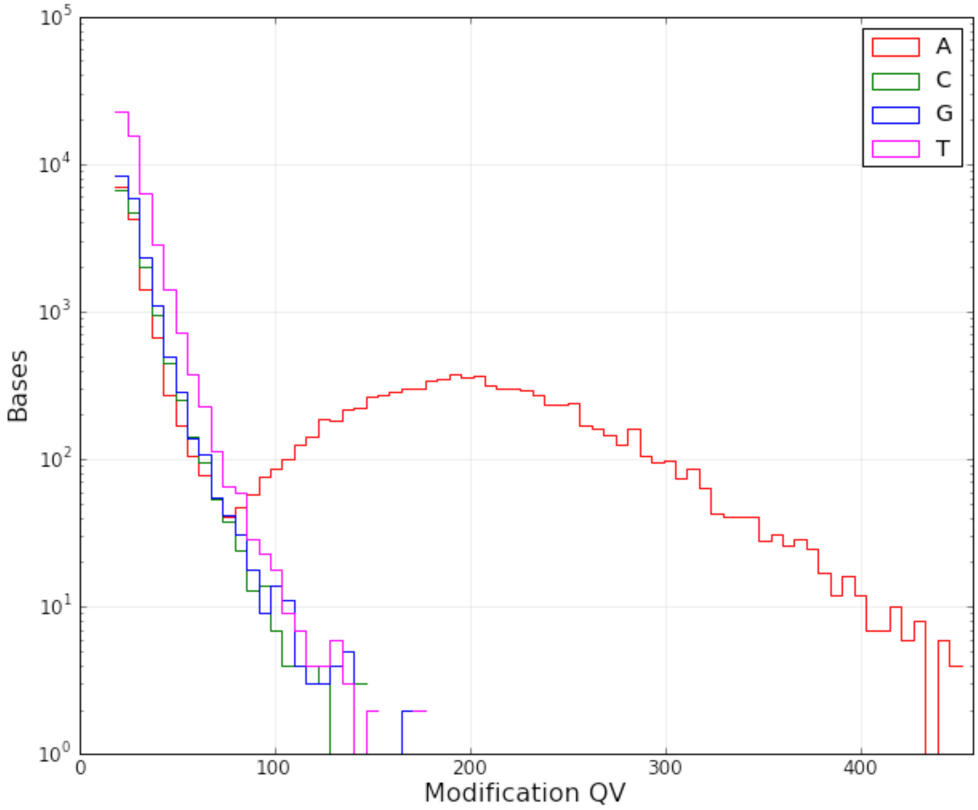

Supplementary Figure 19 – Modification quality value distributions per base. Average quality values for modification of each base in the *Methylacidiphilum fumariolicum* SolV genome are used to estimate the overall distribution and to determine appropriate threshold for reliable identification of methylated bases.

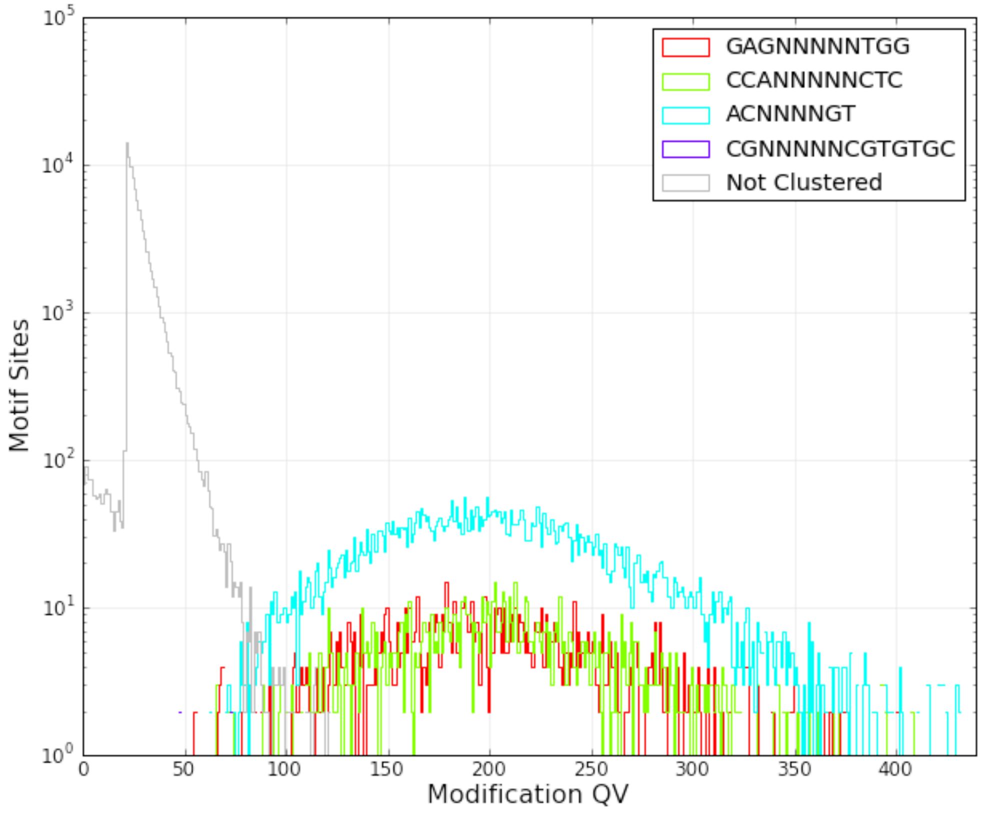

Supplementary Figure 20 – Modification quality value distributions per modification motif per occurrence in the genome of *Methylacidiphilum fumariolicum* SolV.

References:

1. Hou S, Makarova KS, Saw JH, Senin P, Ly BV, Zhou Z, Ren Y, Wang J, Galperin MY, Omelchenko MV *et al*: **Complete genome sequence of the extremely acidophilic methanotroph isolate V4, Methylacidiphilum infernorum, a representative of the bacterial phylum Verrucomicrobia**. *Biology direct* 2008, **3**:26.
2. Krumsiek J, Arnold R, Rattei T: **Gepard: a rapid and sensitive tool for creating dotplots on genome scale**. *Bioinformatics* 2007, **23**(8):1026-1028.
3. Khadem AF, Pol A, Wieczorek AS, Jetten MS, Op den Camp HJ: **Metabolic Regulation of "*Ca. Methylacidiphilum Fumariolicum*" SolV Cells Grown Under Different Nitrogen and Oxygen Limitations**. *Frontiers in microbiology* 2012, **3**:266.
